# Artificial intelligence for tracking social behaviours and supporting an autism spectrum disorder diagnosis: systematic review and meta-analysis

**DOI:** 10.1016/j.ebiom.2025.105931

**Published:** 2025-09-26

**Authors:** Carter Sun, Alistair McEwan, Kelsie A. Boulton, Eleni Andrea Demetriou, Ayesha K. Sadozai, Amit Lampit, Adam J. Guastella

**Affiliations:** a Clinic for Autism and Neurodevelopment (CAN) Research, Brain and Mind Centre, Children's Hospital Westmead Clinical School, Faculty of Medicine and Health, University of Sydney, Australia; b Child Neurodevelopment and Mental Health Team, Brain and Mind Centre, University of Sydney, Australia; c School of Biomedical Engineering, Faculty of Engineering, University of Sydney, Australia; d Department of Psychiatry, University of Melbourne, Melbourne, Australia

**Keywords:** Autism spectrum disorders, Facial information, Social interaction assessment, Systematic review, Meta-analysis, Clinical diagnosis

## Abstract

**Background:**

Artificial intelligence (AI) holds promise for developing tools that can track social behaviours and support clinical assessments and outcomes in Autism Spectrum Disorders (ASD). This review evaluated existing AI algorithms for extracting facial information during social interaction assessments and contributing to diagnostic accuracy for ASD assessment and response to therapy.

**Methods:**

Systematic review of studies on human participants with an ASD diagnosis, sourced from Medline, Embase, Scopus, Web of Science, IEEE Xplore, and ACM Digital Library, evaluated the diagnostic accuracy of AI algorithms in ASD classification and their use in tracking social development through facial information for clinical application in social interactions. Bivariate and multi-level models addressed dependencies, heterogeneity, moderators (modalities, algorithms, tasks), and applied robust variance estimation. Publication bias was evaluated with funnel plots. The QUADAS-2 tool assessed the risk of bias and applicability. This study was registered on PROSPERO (CRD42021249905).

**Findings:**

Of 40,570 studies identified, 38 met the review criteria, and seven provided sufficient data for meta-analysis. The pooled diagnostic odds ratio of 15.917 (95% CI [4.775–53.059]), and bivariate analysis estimated an area under the receiver operating characteristic curve of 0.862. Accuracy improved with facial features, unstructured play, support vector machines, and decision tree-based algorithms. AI methods can analyse social behaviours, including eye gaze on social stimuli, emotional expression, and joint attention in ASD assessments. AI-enabled robots have also been used to guide therapy.

**Interpretation:**

This study shows that AI can accurately and objectively augment ASD assessments, track social behaviours, and enhance therapy outcomes. Further validation in diverse populations is needed to ensure clinical applicability and ethical use.

**Funding:**

None.

**Research in context**

**Evidence before this study**

The potential of data-driven technology in the assessment and intervention of autism spectrum disorders (ASD) is one of the most important current issues for the field. However, given the heterogeneity of ASD behavioural phenotypes described in the Diagnostic and Statistical Manual of Mental Disorders, Fifth Edition (DSM-5) criteria, the development, validation, and integration of algorithm-based methods for objectively assessing and tracking social behaviours have yet to be widely adopted in real-world clinical practice. We searched Medline, Embase, Scopus, Web of Science Core Collections, IEEE Xplore, and ACM Digital Library, from their inception to February 10, 2025, without language restrictions, to identify studies using artificial intelligence-based algorithms for detecting social behaviours and the use of facial behavioural patterns during naturalistic social interaction assessments to support ASD diagnosis. The search terms are centred around “autism”, “artificial intelligence”, “facial”, and “social interaction”. Review articles sourced predominantly focused on social visual attention, emotion recognition, and the accuracy of machine learning algorithms for ASD diagnosis using brain imaging, contributing to a global consensus that facial behavioural markers could be applied in ASD research for diagnosis. For image or video-based analysis, the existing literature focuses on the extraction of facial and gaze movement patterns, and their exploration as early markers. However, factors such as controlled experimental conditions in clinical settings complicate the quantification of human behaviour in real-world practice.

**Added value of this study**

To the best of our knowledge, there has not been a systematic review and meta-analysis to review studies reporting the diagnostic accuracy of artificial intelligence-based methods in performing ASD diagnosis using behavioural phenotypes presented in naturalistic social assessments. We found that artificial intelligence algorithms demonstrated a high diagnostic odds ratio in detecting ASD based on facial and gaze movement patterns in social interaction assessments, with robust results confirmed through sensitivity analysis. Accuracy was higher in unstructured play environments compared to structured diagnostic assessments. Machine learning models, such as support vector machines and decision tree-based algorithms, have been widely explored and showed stronger evidence in supporting ASD diagnosis. Deep learning is more often applied to social behaviour detection by the implementation of robot-assisted intervention programs. Our review emphasises the diversity of experimental designs reported and the importance of reporting comparable data that can be used for clinical decision making.

**Implications of all the available evidence**

This review highlights the promising role of artificial intelligence algorithms in enhancing ASD diagnosis and social marker detection in social tasks, which holds great potential to integrate such technology into clinical practices. Stakeholders (healthcare professionals and policymakers) should prioritise the development of standardised protocols to facilitate the adoption of digital tools and streamline their use in clinical services. Future studies should focus on developing such algorithms within unrestricted social settings, ideally embedding them in standardised clinical assessment tasks, to assess naturalistic responses while also ensuring their ethical compliance in diverse populations.

## Introduction

Autism spectrum disorders (ASD) consist of a range of conditions that influence social communication, reciprocal social interaction, and repetitive behaviour.^1^ Current methods for making a diagnosis of ASD can be time-consuming, resource-intensive, and require a degree of expert clinical skill.^2^ For example, the Autism Diagnostic Observation Schedule-2nd edition (ADOS-2),^3^ is one of the most widely used observational assessments of social interaction and repetitive behaviour. It provides a standardised assessment to support a diagnosis of ASD. Its implementation requires the standardised delivery of social scripts in social interaction, observations of behaviours (e.g., eye gaze, social reciprocity, and joint attention during play), and requires the rater to choose scores based on their observations and memory of complex behaviour. Such ratings may need further debate and deliberation to reach an agreed consensus. Much has been written about the extensive training and support that is required to maintain high-quality standards. It is not surprising, given this process, that reliability in the real-world practice of such methods remains an ongoing concern.^4^^,^^5^ Challenges in objectively tracking such behaviours have also caused concerns about the ability to monitor social behaviours over time, particularly for developmental surveillance,^6^ and for clinical trials.^7^ With an exponential increase in the demand for ASD assessments over the last twenty years,^8^ many assessment services report long delays and/or large fees for service.^9^

Artificial intelligence (AI) may provide solutions to improve access, objectivity, and scalability^10^ in assessments for autism and social behaviour. Previous literature in the autism field have focused on AI applications in clinical reports^11^ and brain imaging.^10^ To date, no systematic evaluation has examined the use of digital AI tools and algorithms to assess and track social behaviours and to inform autism diagnosis. To deepen our understanding of the potential utility of AI in quantifying social behaviour and supporting diagnostic assessments of autism, a synthesis across studies using different types of behavioural data (e.g., facial expressions, gaze, vocal cues, and body movements) is required. This systematic review and meta-analysis aimed to examine existing literature on AI-based behavioural tracking of autism, focussing on algorithms suitable for autism diagnosis in natural social interaction settings, with emphasis on facial features.

## Methods

### Ethics

This systematic review and meta-analysis was based solely on a secondary analysis of aggregated data from previously published studies. No individual-level data were collected or accessed. As such, ethical approval for the review was not required. The included studies generally reported obtaining ethical approval from their respective institutional review boards or ethics committees, and most confirmed that informed consent was obtained from participants or their guardians. The review was registered on PROSPERO with the registration number CRD42021249905.

### Search strategy and selection criteria

This systematic review and meta-analysis adheres to the Preferred Reporting Items for a Systematic Review and Meta-analysis of Diagnostic Test Accuracy Studies (PRISMA-DTA) guidelines,^12^^,^^13^ specifically focused on AI applications for diagnosing individuals with ASD and evaluating meaningful behavioural markers in clinical assessments. Only published peer-reviewed empirical studies with participants formally diagnosed with ASD were included. Studies that involved a control group of typically developing (TD) individuals also had their demographic information extracted, with AI algorithms evaluated using quantitative methods in social interaction settings. Animal studies, as well as those not using AI algorithms (including machine learning or deep learning) for ASD classification or social biomarker recognition in social interaction contexts, and studies that lacked transparency in model training and performance evaluation, were excluded.

The review considered gold-standard diagnostic assessment tools commonly used in diagnostic assessments of autism, such as the Autism Diagnostic Interview-Revised (ADI-R), DSM-5 and ADOS-2,^14^^,^^15^ as eligible reference standards. Studies that did not explicitly report their reference standards but indicated that the diagnosis was conducted by expert clinicians were also included, although this was a minority of studies. The AI algorithms reported in the eligible studies were referred to as index tests. Each reported model prediction result was used to check the performance of the index test against clinical diagnoses using the gold standard diagnostic assessment tool (the reference standards). The eligible studies also need to report their experimental tasks incorporating a social interaction component, as this study is designed to analyse the feasibility and effectiveness of existing technologies in assessing natural social interactions between co-located social partners.

The interactor could be a human, a social robot, or a computer agent (such as video stimuli or an avatar) who attracted the participants' attention through social interaction. Any study design (longitudinal, cross-sectional) in any setting, provided it meets the inclusion criteria with no lower year limit. There were no limitations in terms of years, language, and type of publications to ensure no biased searching and missing articles.

Studies were sourced from Medline, Embase, Scopus, Web of Science Core Collections, IEEE Xplore, and ACM Digital Library. The timeline was set for all articles that meet the search criteria on each database up until the 10th of February 2025. Detailed search terms are presented in Supplementary Table S1. A snowballing approach was conducted when full-text screening of the identified articles was completed. Two reviewers (C.S. and A.K.S.) screened the studies, conducted data extraction, and assessed the risk of bias and applicability, with conflicts resolved by a third independent reviewer (E.A.D.).

### Study selection

The articles found from each database were first imported into EndNote, and duplicates were removed. The remaining studies were then exported to Covidence for further duplicate removal and screening preparation. Two reviewers (C.S. and A.K.S.) screened the study abstract based on the inclusion and exclusion criteria.

In the preliminary screening process, conference papers were filtered if they had no full-text length to maintain the quality and reliability of the included literature, to minimise bias, and to ensure the integrity of the findings. After excluding articles that were irrelevant to the subject of the review, the remaining papers were transferred to full-text review to confirm whether they met the inclusion criteria and select articles suitable for data extraction. The full-text articles were shared with all reviewers through EndNote. Conflicts were resolved by a third independent reviewer (E.A.D.).

During the full-text screening, references cited by the article authors were also reviewed for additional abstract screening. This approach helped minimise the risk of missing eligible studies. Relevant information, such as demographic data from these referenced articles, was also extracted when such details were not reported in the primary study under review. Articles that met the inclusion criteria and reported comparison results of their proposed index tests with their reported reference standards for ASD diagnosis were included in the meta-analysis. The corresponding authors of the eligible article were contacted if the article did not have sufficient data to conduct the meta-analysis. Two authors provided the required data to be considered in the meta-analysis. Those eligible studies that still lack the data needed for meta-analysis were included in the systematic review.

### Data analysis

#### Definitions for data extraction

Data extraction tables were pre-defined to extract the raw data from the included papers. Variables extracted from the eligible studies included: author, year of publication, research focus, population (formal diagnosis of ASD, high-risk group with subsequent confirmed diagnosis of ASD, TD), demographics (country, race and ethnicity), ASD diagnostic tools (reference standards) and other scales used, type of assessment (diagnostic, screening), autism severity level, type of control group, age (mean, standard deviation) or age range, male distribution, sample size, IQ (assessment tools, type and composite scores (mean and standard deviation)). Study design variables include type of experiment, experimental tasks, duration, device used, and interaction type.

To ensure transparency in model training and performance evaluation, detailed information about the type of input data used in the models (also referred to as modalities, such as, facial expressions, vocalisations, head pose, and body movement), the corresponding feature extraction algorithms, type of feature inputs, and type of output classes were extracted. Additional details around model settings are also extracted, which included: datasets used, cross-validation and data augmentation management strategies, classification algorithms (index tests), thresholds, and performance measures (accuracy, precision, sensitivity (or recall), specificity, negative predictive value, positive predictive value, area under the curve (AUC), F1 score, Matthews correlation coefficient (MCC)).

An outcome is considered a true positive (TP) when it has been confirmed by field experts or by a gold standard diagnostic tool, and the proposed index tests successfully predict it. An outcome is defined as a true negative (TN) when confirmed as a comparison group for TP. A false negative (FN) is defined when the actual outcome is positive but predicted as negative. A false positive (FP) is defined as a result when the actual outcome is negative but predicted as positive.

### Assessment of risk of bias and applicability

The Quality Assessment of Diagnostic Accuracy Studies 2 (QUADAS-2) tool^16^ was used to assess the risk of bias and applicability. QUADAS-2 assesses each study in four domains: patient selection, index test, reference standard, and the flow and timing of patients in the study. Judgements of risk of bias and level of concerns were categorised as LOW/HIGH/UNCLEAR, and the tool was also used to help determine the level of risk of bias by completing further detailed sub-questions (Yes/No/Unclear). The total score and details of each assessment question, indicating the risk of bias and applicability concerns, are provided in Supplementary Figure S5 and Supplementary Table S11.

### Synthesis of results

A systematic review of all eligible studies was first conducted. In studies that reported multiple relevant outcomes, such as using different combinations of features as AI model input (for example, facial landmarks, gaze moving patterns), different AI algorithms (index tests) for ASD and TD classification or social behaviour recognition, each unique combination of model, feature, and task extracted and treated as a separate outcome. This approach ensured that all relevant model configurations presented in the original articles were included systematically, without selectively reporting only the best performance results.

The eligible studies can be broadly grouped into binary outcomes (such as ASD or TD, eye contact or without eye contact, attention and distraction) and multiple outcomes (such as performance scores for responses to joint attention, responsiveness scores for assessment tasks, and emotion prediction accuracy). The studies that included binary outcomes with sufficient data (specificity and sensitivity, and contingency table) and designated the classification of ASD and TD are included in the meta-analysis. If a study met the criteria for meta-analysis but lacked contingency table information, data were requested from the corresponding author of the study. In the primary meta-analysis, contingency tables were prepared for the outcomes of each machine learning or deep learning algorithm, along with the corresponding input feature modalities, to calculate proportional data: sensitivity and specificity, and their corresponding 95% confidence intervals (CIs). Sensitivity was defined as the proportion of ASD cases correctly identified by each unique AI algorithm and data modality in the study. Each element of the contingency table was extracted directly from the included studies or provided by the corresponding authors.

### Statistics

The *mada* (version 0.5.11) and *metafor* (version 4.2-0) packages recommended in the Cochrane Handbook for Systematic Reviews of Diagnostic Test Accuracy^17^ were used for quantitative meta-analysis in R (version 4.2.2). First, univariate analyses were performed to evaluate overall sensitivity, specificity, and diagnostic odds ratio (DOR), a combined metric that reflects the performance of AI methods introduced in the literature, differentiating individuals with ASD from the control group. Results were presented in the form of forest plots. Univariate models assume independence between effect sizes. Including multiple outcomes from the same study violates this assumption, and leads to correlated estimates and inflated precision. To address this issue, studies reporting on the same participant group were identified and grouped, and only the best-performing outcome from each group was retained. This ensured that each data point in the univariate meta-analysis represented an independent sample across different research studies.

Second, a bivariate analysis was conducted using the *reitsma* function^18^ to account for the inherent correlation between sensitivity and specificity. Outcomes from studies reporting the same participants were aggregated at the study group level to minimise bias from duplicate sampling. Overall diagnostic accuracy was presented using a Summary Receiver Operating Characteristics (SROC) curve, which plotted the trade-off between sensitivity (true positive rate) and 1-specificity (false positive rate) across all studies. It summarises diagnostic performance aggregated across heterogeneous study settings. The AUC score was used as a measure of overall performance, reflecting how well the AI models performed in comparison to clinical diagnoses. In addition to the qualitative assessment of risk of bias, publication bias was evaluated using the trim-and-fill method on the aggregated data, and visualised with funnel plots to assess asymmetry.

Furthermore, because AI-based analysis compares the effectiveness of different combinations of input feature modalities and algorithm pairs, many studies (or study groups) reported multiple outcomes. A three-level meta-regression analysis was conducted using the *rma.mv* function in the *metafor* package to account for the hierarchical structure of the data, where multiple outcomes were nested within study groups.^19^ The three-level meta-analysis modelled log-transformed DOR, with random effects specified at both the “study group” and “outcome” levels to investigate within- and between-study group variability. Effect sizes were pooled using inverse-variance pooling methods to combine differences in study sample sizes. If any of the values of the contingency table were zero, a continuity correction of 0.5 was applied to avoid zero-counting problems. The between-study variance (τ^2^) was computed using the DerSimonian-Laird estimator, and overall heterogeneity was assessed using Cochran's Q (a statistical test for the presence of heterogeneity), I^2^ (the proportion of variance attributed to each level, calculated using the *var.comp* function), and τ^2^.

Following the three-level meta-analysis, a series of moderator analyses, implemented as subgroup analyses, were conducted using the *rma.mv* function to examine potential sources of heterogeneity observed in the previous three-level meta-analysis. These moderator analyses^20^^,^^21^ were based on categorical study group level variables, including the type of AI models (index tests), input modalities, and experimental tasks. Interactive effects between moderators (index tests and modality, a combination of all three moderators) were also examined. For subgroup estimation, models without intercepts were used to obtain predicted DORs for each category. Individual outcome measures for each study were weighted using the general inverse variance method, and log-transformed DORs were back-transformed for interpretability. Statistical tests of moderator effects included the omnibus test, QM, which assessed whether these moderators (AI models (e.g., Convolutional Neural Network (CNN), Hidden-order Markov Model (HMM), Variable-order Markov Models (VMM), K-nearest neighbour (K-NN), Naïve Bayesian (NB), Random Forest (RF), Support Vector Machines (SVM)), input features (e.g., facial expressions, gaze, head pose, vocal features, posture, posture with recorded images, and their combinations), experimental tasks (e.g., simulated interaction task, still face paradigm)) explained significant variation in effect sizes. The residual heterogeneity test, QE, was also reported to assess any unexplained variation that remained after accounting for these moderators. These analyses accounted for dependency due to repeated outcomes from the same participant groups, consistent with the structure used in the three-level analysis. Robust variance analyses^22^ were conducted using the *robu* function to account for small study effects and dependence from repeated measures within study groups. Sensitivity analyses were conducted using the *sensitivity* function from the *robumeta* package in R to assess the robustness of the meta-regression results across a range of within-study correlation values (rho = 0–1).

### Role of funders

There was no funding source for this study.

## Results

### Study selection and characteristics

**Fig. 1:**
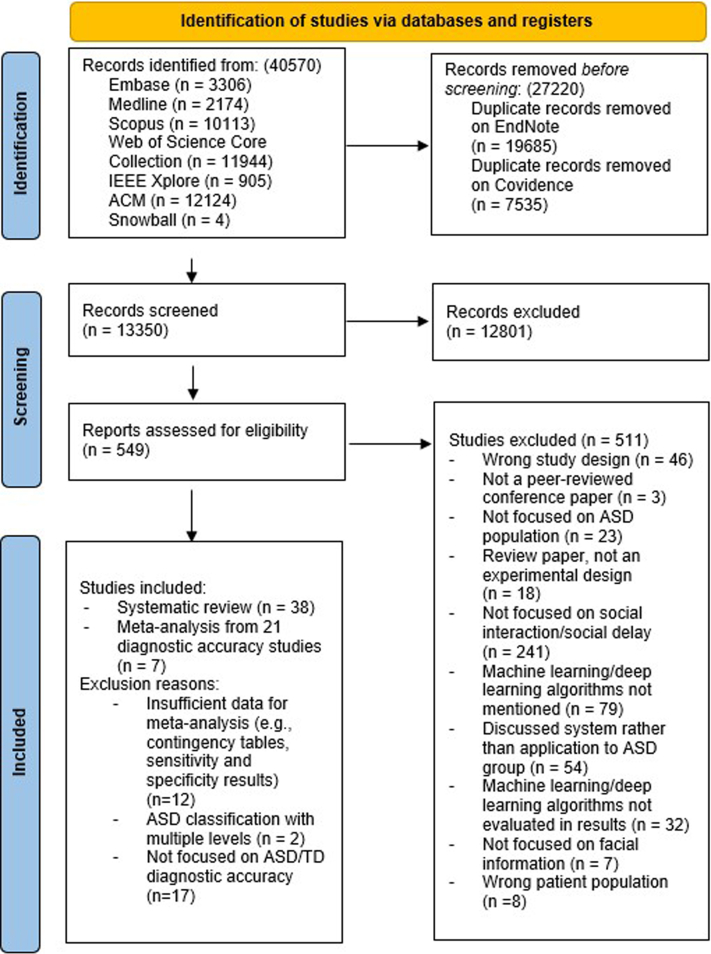
**PRISMA flowchart of literature search strategy**.

A total of 40,570 abstracts were identified, of which 13,350 were screened after removing 27,220 duplicates. There were 12,801 abstracts excluded as they did not meet the inclusion criteria at the abstract screening stage. The remaining 549 full-length articles, including four additional studies found during snowballing, were screened, resulting in 38 studies fulfilling the inclusion criteria for the systematic review. The detailed study selection process is illustrated in Fig. 1.

Table 1 summarises the main study characteristics of each eligible study. A total of 2347 participants (1515 ASD, 1301 TD) were reported in the eligible studies (ages 0–13 years) and two adult groups (up to 50 years). Nine studies did not provide age information. Twenty-eight studies reported reference standards, including gold standard diagnostic tools for an ASD diagnosis, while ten studies did not provide this information. Detailed information on reference standards, type of assessment, and demographic characteristics are provided in Supplementary Table S2. Information on experiment types and durations are summarised in Supplementary Table S3, while Supplementary Table S4 details model selection and performance. Lastly, Supplementary Table S5 provides an overview of the feature extraction algorithms and toolboxes used.

**Table 1:** Summary of study characteristics and algorithm selection strategies in studies included in the systematic review.

Authors	Demographics	Experimental tasks	Interaction type	Type of feature inputs	Machine learning algorithms	Dataset management
**Focus: ASD/TD prediction**						
Tang et al., 2020^23^^,^^a^	Country: China ASD: n = 40; Age: 1.64 ± 0.32 years; Gender: 36M/4F TD: n = 43; Age: 1.37 ± 0.39 years; Gender: 32M/11F	The Still-Face Paradigm	Human-Human Interaction	Head Movement Features, Facial Appearance Features, Vocal Features	SVM, K-NN	Subject-independent 10-fold cross-validation, 90% training and 10% testing Threshold: 0.7–0.8 for sensitivity and specificity, PPV (OR precision) > 0.5 acceptable
Drimalla et al., 2019^24^^,^^a^	Country: Germany ASD: n = 37; Age range: 22 ± 62 years; Gender: 19M/18F TD: n = 44; Age range: 18 ± 49 years; Gender: 22M/22F	Simulated Interaction Task	Human-Human Interaction	Facial Expression, AU Intensity, Gaze Angle, Audio	SVM (radial basial function kernel), Random Forest, Stacked CNN, Pooled CNN	Nested cross-validation (an outer leave-one-out cross-validation loop and an inner 3-fold cross-validation loop for hyperparameters tunning)
Drimalla et al., 2020^25^^,^^a^	Country: Germany ASD: n = 37; Age range: 22 ± 62 years; Gender: 19M/18F TD: n = 43; Age range: 18 ± 49 years; Gender: 21M/22F	Simulated Interaction Task	Human-Human Interaction	Facial Expression Features, Gaze Behaviour Features, Voice Characteristics	Random Forest	Nested cross-validation (an outer leave-one-out cross-validation loop and an inner 3-fold cross-validation loop for hyperparameters tunning) Threshold: 0.5
**Focus: ASD/TD prediction (binary outcomes)**						
Lohan et al., 2018^26^^,^^b^	Country: Scotland ASD: n = 24 (from 31 recruited); Age: 10.72 ± 1.77 years; Gender: 27M/4F (recruited) TD: n = 29 (from 33 recruited); Age: 10.53 ± 1.13 years; Gender: 29M/4F (recruited)	Avatar using “Blende” 2.6 for participants to learn RJA/IJA	Human-Agent Interaction	Pupil Diameter Data, AOI	Sequence to Sequence recurrent neural network (LSTM)	Four-way cross-validation
Alie et al., 2011^27^^,^^a^	Country: United States ASD: n = 6 TD: n = 26 Age range for both groups: 6 months old Gender: not reported	Parents and Children Interaction	Human-Human Interaction	Gaze Pattern	VMM, HMM	Leave-one-subject-out cross-validation
Qiu et al., 2020^28^^,^^a^	Country: China ASD: n = 40 (from 45 recruited); Age: 1.64 ± 0.29 years; Gender: 40M/5F (recruited) TD: n = 43; Age: 1.37 ± 0.39 years; Gender: 32M/11F	The Still-Face Paradigm	Human-Human Interaction	Behavioural Characteristics	SVM, Naïve Bayes, Random Forest	–
Zhang et al., 2022^29^^,^^b^	Country: China ASD: n = 143; Age range: 4–7 years; Gender: 126M/17F TD: n = 113; Age range: 4–7 years; Gender: 91M/22F TD adult: n = 43; Age range: 19–32 years; Gender: 20M/23F	Stimulus Video (Eye-Gaze-Shifting, Gesture Shifting)	Human-Agent Interaction	Valid Sampling Rate, Number of RJA Events	Logistic Regression	10-fold cross-validation
Varma et al., 2022^30^^,^^b^	Country: United States ASD: n = 68; Gender: 53M/15F TD: n = 27; Gender: 18M/9F Age range for both groups: 2–15 years old	Social Gameplay Videos (Semi-Structured)	Human-Human Interaction	Gaze Fixation Patterns (Coordinates), Window And Shift Parameter Values Tuned.	LSTM	Held-out test set. Threshold: Bonferroni-corrected significance threshold of 0.0031
Alcaniz et al., 2022^31^^,^^b^	Country: Spain ASD: n = 35 TD: n = 20 Age range for both groups: 4–7 years old Gender: not reported	VR Static And Dynamic Social And Non-Social Stimuli	Human-Agent Interaction	Number Of AOIs Seen Per Scene On Social And Non-Social Items And Humans	Naïve Bayes, XGBoost, K-NN, Random Forest, and SVM	5-fold cross-validation
Akin-Bulbul et al., 2022^32^^,^^b^	Country: Turkey ASD: n = 61; Gender: 37M/24F TD: n = 72; Gender: 38M/34F Age range for both groups: 26–36 months	Passive Visual Stimuli Social Interaction and Toy Dataset; Passive Visual Stimuli Social Interaction and Animation Dataset	Human-Agent Interaction	Fixation Count, Fixation Duration, Dwell Time, Net Dwell Time, Diversion Duration	Decision Tree, Naïve Bayes, Random Forest, SVM	10-fold cross-validation, Feature selection (Relief, IG, Wrapper)
Liu et al., 2024^33^^,^^b^	Country: China ASD: n = 29; Gender: 20M/9F TD: n = 30; Gender: 20M/10F Age range:5–8 years	Eye Tracking Game	Human-Agent Interaction	Gaze	TCN, Bi-LSTM, GRUA, GNM, TDEA-Net	–
Adham et al., 2022^34^^,^^b^	Country: United States ASD: n = 54; Gender: 44M/10F TD: n = 32; Gender: 15M/17F Age range: recruited participants across all lifespans	A Battery of Eye Tracking Video Stimuli (Activity Monitoring, Social Referencing, Theory of Mind, Dyadic Bid Tasks)	Human-Agent Interaction	Eye Gaze Scan Path (Spatial, Temporal, Gaze Velocity)	DNN, CNN	Train/test ratio (7:3) Threshold: velocity thresholds
Zhao et al., 2021^35^^,^^a^	Country: China ASD: n = 19 from 20 recruited; Gender: 17M/2F TD: n = 20 from 23 recruited; Gender: 17M/3F Age range for both groups: 6–13 years	Structured Conversation	Human-Human Interaction	Gaze Fixation Time (Three Features)	SVM, Linear Discriminant Analysis, Decision Tree, Random Forest	Leave-one-out-cross-validation Threshold: velocity thresholds
Krishnappababu et al., 2021^36^^,^^b^	Country: United States ASD: n = 40; Gender: 31M/9F TD: n = 396; Gender: 194M/202F Age range for both groups: 17–36 months	Recorded 6 Movies with Social and Non-Social Components	Human-Agent Interaction	Integrated Entropy, Positive Energy on Different Movies	–	Leave-one-out-cross-validation
Kojovic et al., 2021^37^^,^^a^	Country: Switzerland ASD: n = 68 + 101, Gender: (40 + 100)M/(28 + 1)F; age range: 1.2–6.9 years TD: n = 68 Gender: 40M/28F; age range: 1.2–5.1 years	Social Interaction Videos (ADOS Assessment)	Human-Human Interaction	Skeletal Information, Video 5 Seconds	VGG16 CNN + LSTM	80–20 train-test split
Minissi et al., 2024^38^^,^^b^	Country: Spain ASD: n = 39; Gender: 32M/7F; Age: 53.14 ± 12.38 months TD: n = 42; Gender: 19M/23F; Age:5 7.88 ± 11.62 months	Virtual reality	Human-Agent Interaction	Posture movement, gaze and response to tasks	Linear Support Vector Classifier	Nested cross-validation
Ko et al., 2023^39^^,^^b^	Country: South Korea ASD: n = 45; Gender: 24M/21F; Age: 48.0 ± 13.4 months TD: n = 50; Gender: 27M/23F; Age: 47.9 ± 12.5 months	ESCS and ABA	Human-Human Interaction	Videos	CNN-LSTM-Attention	10-fold group-wise (by individual) cross-validation Threshold: 0.5
Saakyan et al., 2023^40^^,^^b^	Country: Germany ASD: n = 83; Gender: 48M/33F/2Diverse TD: n = 81; Gender: 40M/41F Age range for both groups: 18–63 years	Simulated Interaction Task	Human-Agent Interaction	Facial Expression features, Voice Features, Gaze, Head movement features	XGBoost	Nested cross-validation Threshold: 0.5
McDonald et al., 2023^41^^,^^b^	Country: United States ASD: n = 15 TD: n = 27 Overall age range: 19.7–49.5 years; Overall Gender: 36M/6F	A modified version of the Contextual Assessment of Social Skills	Human-Human Interaction	Head movement features (monadic, dyadic)	SVM	10-fold cross-validation
**Focus: ASD/TD prediction (multiple outcomes)**						
Zhang et al., 2022^42^	Country: United States ASD: n = 33; Gender: 26M/7F; Age range: 16–37 years	ADOS-2 Interview	Human-Human Interaction	Local Phase Quantisation in Three Orthogonal Planes (LPQ-TOP)	Dictionary learning through sparse coding (K-SVD, k-singular value decomposition) + dimensionality reduction through factor analysis (Marginal Fisher Analysis)	10-fold cross-validation Threshold: 0.75
Megerian et al., 2022^43^	Country: United States ASD: n = 122 from 711 participants with mixed conditions; Gender and Age: unknown for the ASD group	Questionnaire + two social interaction videos	Human-Human Interaction	–	–	Threshold: PPV greater than 65% and NPV greater than 85%
**Focus: attention or eye contact detection**						
Di Nuovo et al., 2018^44^	Country: Italy ASD: n = 6; Gender: 6M; Age range: 66–121 months	VB-MAPP Imitation tasks	Human-Robot Interaction	Face Detection, Facial Landmark	Naïve Classification, K-NN Classifier	10-fold cross-validation
Eunji et al., 2017^45^	Country: United States ASD: n = 50 TD: n = 50 Overall age range: 1.6–13.7 years; Overall Gender: 74M/26F	Semi-Structured Play Interaction	Human-Human Interaction	Eye Contact Detection	PiCNN, AlexNet, PEEC, GazeLocking	5-fold cross-validation, 80% training, 20% testing for each disjoint train/test splits Threshold: 0.9
Chong et al., 2020^46^	Country: United States ASD: n = 66; Gender: 55M/11F; Age: 44.0 ± 11.11 months TD: n = 55; Gender: 36M/19F; Age: 27.45 ± 5.84 months	ESCS and BOSCC	Human-Human Interaction	Cropped Face Regions	Deep Learning (ResNet-50 backbone)	Threshold: 0.9
**Focus: gaze shift**						
Chong et al., 2017^47^	Country: United States ASD: n = 8; Gender: 4M/4F; age range: 32–60 months TD: n = 8; Gender: 5M/3F; age range: 20–36 months	ESCS	Human-Human Interaction	Gaze Shift	SVM	20% of samples trained
Zhang et al., 2021^48^	Country: United States ASD: n = 10; Gender: 7M/3F; Age range: 5–12 years	Standard Therapy: table-top reading activity, play therapy: drumming activity, singing activity	Human-Human Interaction	Prediction Scores and Ground Truth by Experts	Deep CNN (Three branches)	Pretrained on AVA/UCO-LAEO, AFLW datasets Threshold: mutual gaze score (0.6)
**Focus: joint attention performance level and interaction type classification**						
Nie et al., 2018^49^	Country: United States ASD: n = 34; Gender: not reported; Age: 2.66 ± 0.54 years	ESCS (RJA part)	Human-Robot Interaction	GMM Of Head Pose, Hard Histogram Of Head Pose, Soft Histogram Of Head Pose, Hard Histogram Of Head Pose Motion, Soft Histogram Of Head Pose Motion	Transductive Support Vector Machine (TSVM) with radial basis function kernel	–
Zhao et al., 2024^50^	Country: United States ASD: n = 83; Age range: 1–12 years; Gender: not reported	Family Observation Schedule-Second Version (FOS-II)	Human-Human Interaction	Image	SlowFast + BERT CNNs	–
**Focus: responsiveness score**						
Liu et al., 2017^51^	Country: United States ASD: n = 22; gender: 16M6F TD: n = 21; gender: 19M/2F Overall age range: 2–3 years	Response to Name	Human-Human Interaction	Latency, Weighted Duration, Latency + Weighted Duration	Decision Tree	–
**Focus: social engagement**						
Anagnostopoulou et al., 2021^52^	Country: Greece ASD: n = 7; Gender: 5M/2F; mean age: 10.6 years TD: n = 25; mean age: 8.6 years; Gender: not reported	Play with robots (Show me the gesture, express the feeling, pantomime, guess the object and joint attention)	Human-Robot Interaction	Pose Features	Alexnet, 2D CNN, LSTM-based RNN, 1D multi-channel CNN	Train/validation, data augmentation (flip vertically with 0.3 probability, add gaussian noise with 0.5 probability)
Javed et al., 2020^53^	Country: United States ASD: n = 5; Gender: 5M; Age: 8.2 ± 1.1 years TD: n = 13; Gender: 8M/5F; Age: 7.07 ± 2.56 years	Social Engagement Estimation (Social interaction included)	Human-Robot Interaction	Movement + Expression	CNN, SVM, Random Forest, Decision Tree, K-NN	10-fold cross-validation (train/test split of 0.8/0.2)
Prakash et al., 2023^54^	Country: India ASD: n = 300, age range: 1–5 years; Gender: not reported	Applied Behavioural Analysis (ABA) intervention	Human-Human Interaction	Videos	Faster-RCNN and Resnet-50	–
Wang et al., 2024^55^	Country: China ASD: n = 13; Gender: 6M/7F, mean age: 28.8 months TD: n = 11; age and gender for TD group not reported	Express Needs with index–finger pointing	Human-Human Interaction	Facial, Posture, Gaze	Feature Clustering Network + Multi-head Attention Network + Attention Fusion Network, MMPose +Yolo, Restnet-18	–
**Focus: facial behaviour and emotion recognition**						
Wu et al., 2021^56^	Country: United States ASD: n = 133 at risk (high/low); Age range: 6–36 months; Gender: Not provided	Adult-child play task	Human-Human Interaction	Image, Facial Features	ResNet-18	Horizontal flip
Li et al., 2021^57^	Country: United States ASD: n = 6; Age range: 5–13 years; Gender: not provided	Therapeutic games for children to play (ASD-affect dataset)	Human-Human Interaction	Facial and Speech Emotion	ResNet-18	Data augmentation and 5-fold cross-validation Threshold: 0.5
Liu et al., 2023^58^	Country: China ASD: n = 16; Age range: 12–36 months; Gender: not provided	Social Interaction With Clinician	Human-Human Interaction	Facial and Skeleton Feature from Images and videos	CNN + Spatial-Temporal Graph Convolutional Network	8-fold cross-validation Data augmentation by trimming long videos into segments
**Focus on head movement**						
Krishnappababu et al., 2023a^59^	Country: United States ASD: n = 41 Gender: 29M/12F; Age: 24.38 ± 4.73 months TD: n = 416; Gender: 209M/207F; Age: 20.59 ± 3.18 years	Social and Non-social Movies with visual and auditory stimuli	Human-Agent Interaction	Images and Gaze	SVM	Leave-one-out cross-validation
Krishnappababu et al., 2023b^60^	Country: United States ASD: n = 41 Gender: 29M/12F; Age: 24.38 ± 4.73 months TD: n = 416; Gender: 209M/207F; Age: 20.59 ± 3.18 years	Social and Non-social Movies with visual and auditory stimuli	Human-Agent Interaction	Total time facing forward, mean blink rate during social task, percentage of time the child gazed at the social elements	Linear Logistic Regression	Leave-one-out cross-validation

IJA, initiation of joint attention; RJA, response to joint attention; AOI, areas of interests; SVM, support vector machine; LSTM, Long Short-Term Memory; VMM, Variable-order Markov Models; HMM, Hidden-order Markov Model; K-NN, K-nearest neighbour; TCN, Temporal Convolutional Networks; GRUA, Gated Recurrent Unit with Attention; GNM, GooLeNet with SVM; PPV, positive predictive value; NPV, negative predictive value; AU, action units.

a Studies provided sufficient data for meta-analysis.

b Studies excluded from meta-analysis due to insufficient data (contingency table and/or performance measures (sensitivity/specificity)); Other studies were focused on social behaviour detection, behavioural performance assessment or ASD severity level prediction.

**Fig. 2:**
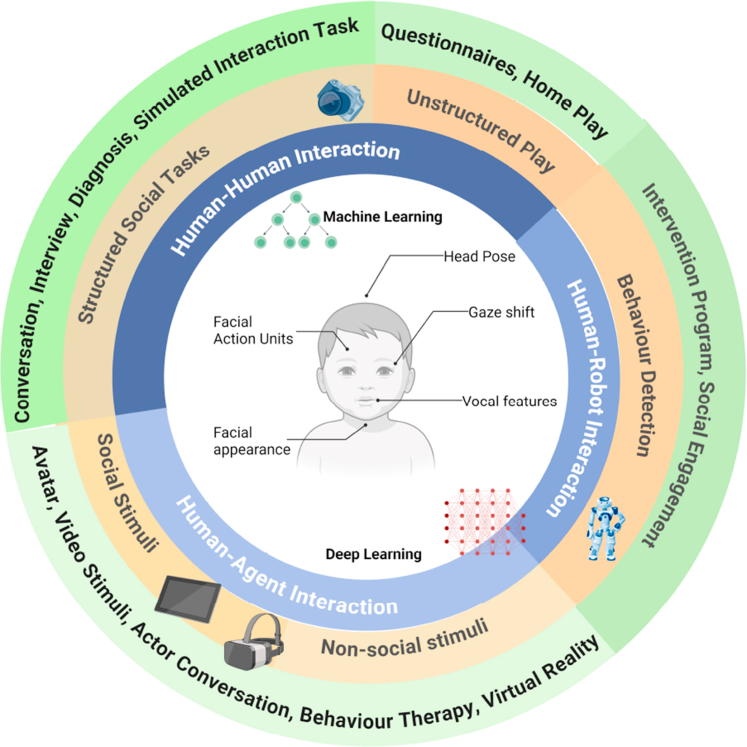
**Overview of AI-driven analysis of social interactions in autism assessments**.

For studies that met the inclusion and exclusion criteria, twenty focused on using AI algorithms and digitalised features to distinguish individuals with ASD from TD groups in social interaction assessments (see Supplementary Figure S1a and c), of which seven studies accumulated a total of 32 contingency table samples for the synthesis of the main effect suitable for a meta-analysis of diagnostic test accuracy (see Supplementary Table S6). The remaining studies were excluded from the diagnosis accuracy meta-analysis because neither the sensitivity and specificity nor the two-by-two contingency table were provided. These samples were drawn from five different human-to-human interaction experiments (Still-Face paradigm, Simulated Interaction Task, Face-to-Face Interaction, Structured Face-to-Face Conversation, and the ADOS-2 Diagnostic Assessment). They contained 383 individuals diagnosed with ASD and 277 from the TD group. Eighteen studies reported their use in behaviour recognition (see Supplementary Figure S1b and d). Outcomes were compared with human-human interaction, human-agent interaction, and human-robot interaction (Fig. 2).

### Systematic review of social interaction assessments in naturalistic settings

#### Structured social interaction between humans

Studies examining AI algorithms applied to structured assessment tasks analysed naturalistic behaviours during recorded interactions, such as facial expressions, head and body movements, and vocal characteristics. Tang and colleagues^23^ used head movement, facial appearances, and vocal features extracted from a 2-min “mother amused child without touch of body” session and a 1-min still-face paradigm to discriminate infants at high risk of ASD from TD. Machine learning algorithms applied in the study were K-NN and SVM. Qiu and colleagues^28^ used the same still-face paradigm and applied children's behaviour characteristics to NB, RF, and SVM algorithms. Kojovic and colleagues^37^ video recorded each ADOS-2 assessment administered in their study, applied a VGG-16-based convolutional neural network to extract pose features, and opted for the Long Short-Term Memory (LSTM) architecture for ASD classification. Similarly, Zhang and colleagues^42^ analysed video recordings of participants aged 16–37 years receiving ADOS-2 interviews for ASD diagnosis. A discriminative few-shot learning approach was used in their study to extract facial information (facial appearance, static expression, and eye movements) from interview scenes to derive predictions of ASD classification and to match the classification into three categories (autism, autism spectrum, and non-spectrum), in line with the ADOS-2 assessment scheme. Ko and colleagues^39^ developed a deep learning model (ResNet18-LSTM-Attention) for ASD classification and severity predictions using video recordings collected during a 10-min Early Social Communication Scales (ESCS) and an Applied Behavioural Analysis (ABA) intervention session. Other studies designed custom tasks to assess social behaviours and for diagnosis.^61^^,^^62^ Zhao and colleagues^35^ conducted structured conversation tasks between an interviewer and the child. They analysed quantifiable features (e.g., gaze fixation duration on areas of interest (AOIs)), and duration of conversation tasks, for use in model training. Algorithms utilised include linear discriminant analysis (LDA), decision tree (DT), RF, and SVM for ASD classification. Drimalla and colleagues^24^^,^^25^ introduced a simulated interaction task to extract ASD-related social markers (facial expression, gaze, and voice) for ASD classification and compared the performance of RF, SVM and CNN models. Alie and colleagues^27^ proposed a 3-min parent-child face-to-face interaction task, where Markov models were applied to analyse gaze patterns of infants to identify those at risk of ASD.

#### Unstructured social interaction between humans

Studies explored the potential of ASD diagnoses using video recordings collected in non-clinical and unstructured home play environments. Gaze-moving patterns and questionnaires were the primary sources of information used to develop AI tools. Varma and colleagues^30^ used gaze fixation and scan-path features applied on an LSTM-based neural network model to obtain preliminary results of 60% accuracy. Megerian and colleagues^43^ suggested a medical device in a multi-site cohort clinical trial providing preliminary ASD screening as a reference for healthcare providers. Their studies involved analysing caregiver and healthcare provider questionnaires and home-taken videos. Participants were classified using a gradient-boosted decision tree model into categories corresponding to people with autism, people without autism, or indeterminate classifications. McDonald and colleagues^41^ analysed monadic and dyadic head movement features from video recordings of 3-min face-to-face conversation sessions using a modified version of the Contextual Assessment of Social Skills. They applied an SVM classifier to predict ASD and TD groups. Liu and colleagues^58^ proposed a deep learning framework that integrates traditional CNN models with spatial and temporal graph convolutional networks to recognise emotions expressed by toddlers based on recorded facial and skeleton features. Zhao and colleagues^50^ applied SlowFast and BERT CNN models to detect social interaction styles during child-parent interactions.

#### Human-agent interaction

The integration of human-agent interaction with the incorporation of external devices and avatar animations in clinical assessments has gained significant attention.^63^^,^^64^ These studies utilised eye gaze, facial dynamics, and responses to stimuli, applying models like logistic regression, machine learning, and deep learning techniques for ASD classification and behaviour therapy. Zhang and colleagues^29^ proposed a logistic regression model for ASD classification based on participants' spontaneous responses to video stimuli containing scenes of eye gaze and gesture-shifting. Akin-Bulbul and colleagues^32^ showed that conventional machine learning algorithms, including DT, NB, RF, and SVM, could be trained using eye gaze features extracted from social (conversation, play between actor and toddler) and non-social (toys or animations) videos for ASD screening. Atyabi and colleagues^34^ and Liu and colleagues^33^ used a collection of eye-gaze scan paths, temporal information, and pupil velocity features trained on CNN and Deep Neural network (DNN) models for ASD classification. They included video tasks such as Activity Monitoring, Social Referencing, Theory of Mind, and Dyadic Bid. Krishnappa-Babu and colleagues^36^^,^^59^^,^^55^ introduced both social and non-social movie tasks and showed atypical facial dynamics, facial expressions, and head movement patterns assisted ASD classification using DT and SVM algorithms. Alcaniz and colleagues^31^ and Minissi and colleagues^60^ proposed that virtual reality (VR) containing static and dynamic social and non-social stimuli could be an assessment tool for ASD diagnosis. They examined the prediction performance of gaze features recorded during VR play using conventional machine learning algorithms such as SVM, RF, NB, XGBoost and K-NN. Lohan and colleagues^26^ suggested that children's eye gaze movements, guided by avatars, could be used to train responses to joint attention and differentiate individuals diagnosed with ASD from individuals with TD using the LSTM model.

#### Human-robot interaction

Research quantified social behaviours by analysing facial and posture features extracted through sensors on the robot, using deep neural network models. Most human-robot interaction tasks were designed to assess social behaviours or as part of intervention programs, leveraging the programmability of robots. The unique appearance and interactive capabilities of robots were often used to attract the patient's attention.

### Systematic review on behaviour detection studies in autism research

#### Facial and gaze behaviour analysis

Visual attention and distraction behaviours could be automatically recognised using trained models through facial emotion and movement patterns recorded in social contexts. For example, in a human-robot therapy session by Nuovo and colleagues,^44^ the softbank robotics, NAO, uses NB and K-NN models to capture facial images of participants during imitation tasks in the Verbal Behaviour Milestones Assessment and Placement Program (VB-MAPP) to assess their attention and training performance. Chong and colleagues^45^^,^^46^ conducted a wide range of naturalistic social tasks to detect eye contact behaviours during adult-child interactions. The adult assessor captures facial images of the child participant using wearable cameras embedded in glasses. Deep learning models such as ResNet-50, AlexNet, Pose-implicit Convolutional Neural Networks (PiCNN), Pose-Dependent Egocentric Eye Contact (PEEC) detector and GazeLocking were applied for the detection of eye contact moments when the child participants were looking into the wearable glasses at the adult. Chong and colleagues^47^ also proposed an SVM classifier to predict gaze shifts that initiate joint attention behaviour through the obtained head pose and camera pose estimation features. Guo and colleagues^48^ proposed a deep CNN model to score mutual gaze for each detected head pose between child-trainer pairs in ASD therapy training.

#### Social engagement analysis

Studies have examined participants' engagement in receiving therapies in the context of human-robot interaction. Javed and colleagues^53^ proposed a deep CNN model in comparison to conventional machine learning models (SVM, RF, DT, K-NN) to detect social engagement behaviours presented in both ASD and TD groups through the skeleton posture and facial expression features. The engagement behaviours analysed in their study include gaze, smile, triadic interaction, vocalisation, imitation, and self-initiated interaction. Anagnostopoulou and colleagues^52^ proposed deep learning models (1D CNN, ResNet-50, LSTM-based RNN, 2D CNN, AlexNet) to estimate engagement levels based on posture key points detected in social play therapy video recordings. Wang and colleagues^55^ proposed deep learning models (a feature clustering network and a multi-head attention network) to assess social engagement based on facial, posture and gaze features extracted during child pointing behaviour towards bubble bottle.

#### Multi-modal approaches to understanding complex social behaviour

Recognising complex social behaviours has been examined through multi-modal feature learning, utilising a range of data types, such as, facial expression, head poses, emotion response analysis, and applied AI algorithms to enhance ASD assessment. For example, Liu and colleagues^51^ introduced a “response-to-name” dataset in which clinicians rated child responses to a name call during toy play. Through a collection of face detection, head pose estimation, and response latency and weighted duration information, their DT models were able to predict social responsiveness scores. Here, the TD group responded more strongly than the ASD group, and the responsiveness score in the ASD group was lower. Prakash and colleagues^54^ developed a model to assess joint attention, emotion regulation, and human poses based on facial and movement features extracted from deep learning models, such as Faster-RCNN and ResNet-50 for facial feature extraction, and the object detector YOLO-v3, using video recordings collected during ABA therapy sessions. Nie and colleagues^49^ proposed that head pose features (such as hard and soft histograms of head pose data, head pose on different areas of interest extracted by Gaussian Mixture Model, and head pose motion) trained on transductive SVM models with radial basis function kernel enhance the prediction of response to joint attention performance in interpersonal interaction. They suggested that the ESCS test conducted during a human-robot interaction could help predict performance during human-to-human social contexts. Li and colleagues^57^ suggested that facial emotion features extracted from the “ASD-Affect” dataset combined with speech emotion recognition improved the prediction of affect states (positive, negative, and neutral) of children with ASD. Wu and colleagues^56^ proposed a ResNet-18-based deep learning model to identify behaviours in infants associated with ASD diagnosis using video recordings of adult-child interaction. They focused on facial features, such as face and eye landmarks, facial action units, head pose, and gaze direction extracted by the OpenFace2.0 library.

### Risk of bias and applicability

In addition to synthesising the characteristics of included studies, we assessed their quality and applicability using the QUADAS-2 checklist. Overall, 26 studies had a low risk of bias in patient selection due to a clear description of patient enrolment, inclusion, and exclusion criteria. Three studies were categorised as unclear due to a lack of patient selection description. Nine studies were classified as having a high risk of bias due to highly imbalanced participant numbers or because participants were initially classified as having high-risk ASD and later formally diagnosed with ASD or other disorders (non-ASD).

In addition, 38 studies were rated as low risk of bias for flow and timing domains, and 37 studies for index test domains on the QUADAS-2 checklist due to clear reporting of AI models and model training procedures. One study was rated as unclear for the index test, due to a lack of relevant model training information. Since model training occurs post data collection, strict timing requirements were not necessary. Twenty-nine studies reported reference standards and were classified as low risk of bias. Eight studies lacked detailed reference standard descriptions at enrolment, and one study did not mention their used diagnostic tool.

To assess applicability concerns, one study reported participants at low or high risk of ASD without confirmation at enrolment, and another study did not specify age information, resulting in an unclear rating for patient applicability to the review question. The remaining 36 studies had a low risk of patient selection. All studies were rated as low risk for the index test domain, as the AI models applied were reported to be consistent with the review question. Thirty-five studies were rated as low risk for reference standards, while three were classified as unclear due to insufficient information to confirm their applicability. The QUADAS-2 quality assessments are presented in Supplementary Figure S5, Supplementary Table S11.

### Meta-analysis of diagnostic accuracy in AI-based ASD classification

**Fig. 3:**
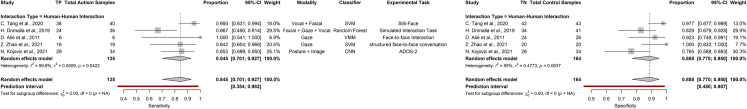
**Univariate meta-analysis of pooled sensitivity and specificity, based on the best diagnostic outcome from each study group. TP refers to the number of autism samples correctly predicted by AI models. TN is the number of control samples correctly predicted by AI models. I^2^ represents the percentage of total variance across study groups that is due to heterogeneity rather than chance. τ^2^ represents the between-study variance in a random-effects model indicating the variance of the true effect size across study groups. The grey shaded box illustrates the sensitivity (left) or specificity (right) along with the 95% CI. The diamond represents and an average performance with 95% CI**.

The uni-variate meta-analyses (Fig. 3) revealed a significant pooled sensitivity of 0.845 (95% CI [0.701–0.927], prediction interval 0.281–0.987, I^2^ = 60%, τ^2^ = 0.501, Q(4) = 9.895, p = 0.042), indicating moderate heterogeneity across studies; and a significant pooled specificity of 0.888 (95% CI [0.770–0.950], prediction interval 0.370–0.991, I^2^ = 55%, τ^2^ = 0.477, Q(4) = 8.899, p = 0.064), indicating that AI models could effectively discriminate between ASD and TD groups. Furthermore, Supplementary Figure S2 revealed a significant pooled effect on the diagnostic odds ratio (DOR) of 58.965 (95% CI [10.889–319.309], p < 0.01, I^2^ = 70.820%, τ^2^ = 2.510, Q(4) = 13.708, p < 0.01).

**Fig. 4:**
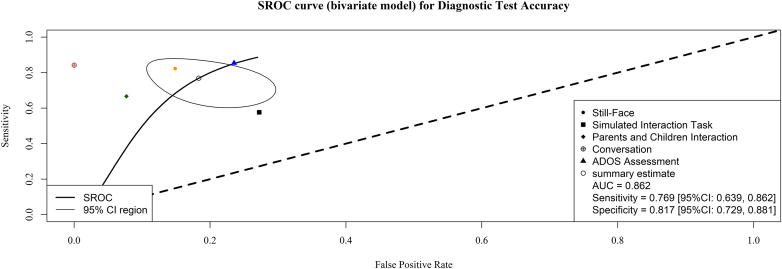
**Summary receiver operating characteristics curve of the studies eligible for meta-analysis aggregated by study groups. The contour represents the 95% CI of the summary estimate of the diagnostic test accuracy. The diagonal dashed line indicates a cut-off threshold of 50% diagnostic test accuracy**.

A bivariate SROC curve (Fig. 4) demonstrated a high pooled sensitivity and specificity of 0.769 (95% CI [0.639–0.862]) and 0.817 (95% CI [0.729–0.881]), respectively, with an AUC of 0.862. A random-effects meta-analysis of the aggregated data (n = 5 study groups) revealed a significant overall effect of 6.240 (95% CI [1.391, 27.979], SE = 0.766, z = 2.392, p = 0.017, prediction interval 0.223–174.347). There was substantial heterogeneity among study groups (τ^2^ = 2.301, SE = 2.055, I^2^ = 87.9%, Q(4) = 25.012, p < 0.0001). Funnel plot asymmetry was detected; however, due to the small number of study groups, Egger's test was not conducted. A trim and fill analysis imputed zero missing outcomes, and the bias-corrected estimates of the true effect were unchanged (Supplementary Figure S3b).

A three-level meta-analysis was conducted to account for the dependency among multiple outcomes within study groups. The overall effect was statistically significant, with a diagnostic odds ratio of 15.917 (95% CI [4.775, 53.059], prediction interval 0.691–366.767, p < 0.0001). Significant heterogeneity was observed (Q(31) = 186.418, p < 0.0001), with notable between-study group variance (τ^2^ = 1.104, n = 5 study groups) and within-group variance (τ^2^ = 1.081, k = 32 outcomes).

The moderator analyses suggested that heterogeneity observed across studies (n = 5, k = 32) was influenced by a combination of 1) classification algorithms used for model training, 2) the diverse input feature modalities learnt by each model, and 3) experimental tasks. No significant moderator effect was found for the classification algorithms alone (QM(6) = 6.305, p = 0.390), although a significant test for residual heterogeneity were found (QE(25) = 127.563, p < 0.0001, between-study group variance (τ^2^ = 1.138, n = 5) and within-group variance (τ^2^ = 1.114, k = 32)). In comparison to the CNN model, only the SVM classifier was found to have significantly improved diagnostic accuracy; other models did not show a significant contribution (Supplementary Table S7). Significant moderator effects were observed on the data modalities used for training AI models (QM(10) = 52.508, p < 0.0001; QE(21) = 56.737, p < 0.0001, between-study group variance (τ^2^ = 2.158, n = 5) and within-group variance (τ^2^ = 0.197, k = 32)), with facial features prominently driving this effect. Other modalities, such as body posture features, and video-recorded images, did not significantly contribute to the moderator effect. Notably, head pose features only was significantly less effective than modalities including facial features (Supplementary Table S8).

Further exploration of moderator interactions, particularly combining the classification algorithms and modalities as a stand-alone moderator (Supplementary Table S9), explained most of the observed heterogeneity across study groups (QM(16) = 105.778, p < 0.0001; QE(15) = 24.582, p = 0.056, between-study group variance (τ^2^ = 4.111, n = 5) and within-group variance (τ^2^ = 0, k = 32)). When examining the types of experimental tasks as part of the moderator interactions, a significant moderator effect persisted (QM(18) = 177.316, p < 0.0001). However, most of the variations were explained (QE(13) = 6.501, p = 0.926, between-study group variance (τ^2^ = 0.001, n = 5) and within-group variance (τ^2^ = 0, k = 32)). Several input data modalities and classifiers used in the included studies did not show a statistically significant difference in predictive performance compared to facial features alone. These modalities include vocal, posture, posture combined with images, gaze-only, head pose combined with vocal, head pose combined with facial features, and the combination of facial, gaze and vocal features. Similarly, classification algorithms, such as HMM, K-NN, and VMM, and the implementation of the still-face paradigm task, were not significantly different from facial features alone. Detailed moderator effects are shown in Supplementary Table S10.

The robust variance estimation and sensitivity analysis revealed that the model assumptions of the main analysis were robust (estimate = 3.018, standard error = 0.617, I^2^ = 79.839%; τ^2^ = 2.313–2.399 as rho increases from 0 to 1).

## Discussion

This systematic review examined studies on the use of AI in social interaction assessment for autism in different settings, where facial features served as a key source of information in ASD research. The results showed high diagnostic accuracy for the AI algorithms to detect ASD, with pooled estimates showing an AUC of 86.2%, sensitivity of 76.9%, and specificity of 81.7%. These findings suggest that AI models trained on facial and gaze movement features can effectively distinguish between individuals diagnosed with ASD and neurotypical controls in social interaction assessments. If these results were replicated in real-world settings, such results would suggest similar clinical utility to other established clinical diagnostic assessment tools. Interestingly, recent research findings supported the utility of facial features in the diagnosis of ASD, as increased attention to facial features was associated with improved diagnostic accuracy. Accuracy was also enhanced in unstructured play environments compared to structured tasks (e.g., ADOS tasks) or human-to-robot interactions. Specific AI algorithms (including SVM and Decision Trees) showed a stronger evidence base in assisting ASD diagnosis. AI algorithms could detect a range of social behaviours, including eye gaze, emotional expression, and joint attention, suggesting opportunities for tracking changes or evaluating responses in clinical trials. Preliminary research has also explored embedding AI algorithms in guiding therapeutic responses and using AI-enabled robots in therapy. These findings highlight the potential for AI to augment clinical ASD diagnostics, track social behaviours, and enhance therapeutic interventions.

Findings suggest the potential of AI-driven algorithms to improve objectivity in the diagnosis and behavioural assessments of ASD. Current clinical diagnosis processes heavily rely on subjective observations of naturalistic social responses, with eye and facial movements serving as critical but difficult-to-code sources of information.^65^ Current studies have successfully utilised facial expressions, gaze moving patterns, and head poses extracted from social interaction tasks to train machine learning algorithms, mostly SVM and Decision Trees, to excel in analysing regular patterns from limited clinical samples. These facial features can be detected using off-the-shelf camera recording systems and eye trackers, scalable and accessible across settings for supporting the detection of behavioural markers for routine monitoring, and aiding clinical assessment in controlled, remote, or home settings. The bivariate meta-analysis evaluating sensitivity and specificity found facial features from still-face paradigms or conversations more stable than complex interactions such as parent-child interaction and structured ADOS assessments, likely due to unique movement pattern derivation in facial modalities by machine learning. Complex behaviour analysis requires advanced deep learning algorithms and intensive computational resources, and often irregular movement patterns add to the modelling complexity.

SVM and Decision Tree machine-learning-based algorithms showed robust evidence, but their use may reflect the earlier phase in the development of such approaches in the field. In human-human interaction studies, ResNet-based deep neural networks and Decision Tree models were predominantly investigated for behaviour detection rather than diagnostic outcomes, with ResNet demonstrating better performance. Notably, facial and gaze features were also commonly used in traditional machine learning algorithms for ASD classification in human-to-computer agent interaction scenarios, such as interaction with avatars or video stimuli. Few studies explored the use of advanced deep learning algorithms in ASD classification during social dyads, with human-to-social robot interaction primarily enhancing therapy sessions to improve social skills. Limited research studied their direct application in ASD and TD classifications. In robot-assisted studies, advanced deep learning algorithms, such as ResNet and AlexNet-based models, and traditional machine learning models, such as K-NN and Random Forest, have been shown to outperform SVM. These robots were able to automatically detect eye contact scenes and facial features, including facial landmarks and head pose.

Despite the potential of AI to support clinical practice, this field remains in relative infancy in terms of co-design and developing ethical guidelines regarding use. This is particularly important considering some of the complex issues that might emerge from using AI generated data to support diagnoses. While data security and privacy are of paramount importance and are often the focus of concern regarding data sharing and storage issues, researchers still have the opportunity to guide the complex discussions about the implementation of such tools in clinical practice^66^ to safeguard against poor practices that could lead to harm.^67^ Furthermore, it is crucial to address the potential dangers of using AI as a replacement for traditional clinical services. Standards and guidance must encourage inclusiveness in diverse populations (gender, age, ethnicity) with a deep understanding of the clinical complexities in populations where behavioural phenotypes differ (impact of biological gender, masking, age, intellectual comorbidities).^67^ Drawing from the literature on ethical AI, transparency and accountability with stakeholders, and continuous monitoring of the impact and trust in appropriate assessments are essential to enable the responsible development of AI tools.

### Study limitations

Although the meta-analysis results are considered robust, further validation is essential. This review extracted additional information, including diagnostic criteria, age, ethnicity and IQ; however, there was insufficient sample to investigate their moderator effects. We acknowledge that race and ethnicity are sociocultural constructs, and differences in algorithm performance across groups may reflect unmeasured confounding factors, such as socioeconomic status or technology accessibility. In addition, there is a notable lack of validation studies involving experts (e.g., clinicians) and limited reporting of associated clinical measures, as well as applications of AI in clinical settings across diverse neurodevelopmental, physical and psychiatric diagnostic populations. Furthermore, not all diagnosis-focused studies were included in the analysis due to missing information, such as sensitivity, specificity, and a contingency table required to conduct a meta-analysis. Only two study authors provided these data upon request.

We also acknowledge heterogeneity of study aims. Our primary goal was to summarise the facial features and AI methods currently applied in autism research, including studies focused on either behavioural tracking or diagnostic classification accuracy. To ensure consistency, we included only studies reporting their use of AI for diagnostic purposes into the meta-analysis component. For studies focused on the use of AI in behavioural analysis, they are discussed in the systematic review sections only. While the systematic review provides a needed summary of the data to date regarding social behaviour outcomes, the diversity in outcomes did not permit us to aggregate this data into a meta-analysis. Moreover, few studies applied cross-validation and data augmentation methods to reinforce the generalisability of trained models and address data imbalances. These methods should be more widely adopted in future studies to prevent exaggerating the performance of the models. We define transparency in model training and evaluation as the clear reporting of validation methods (e.g., cross-validation with dataset partitioning, thresholding, clinical or external validation where possible), minimum information on performance metrics (e.g., contingency table, sensitivity, specificity), use of data augmentation, and computational resources requirements.

We note that the reuse of standardised research cohorts to evaluate multiple algorithms can present important methodological challenges, particularly related to the lack of independence across analyses. Despite these limitations, this practice is important and widely accepted in the AI and healthcare communities,^68^^,^^69^ as it ensures reproducibility and enhances benchmarking, collaboration and research development. In this study, we used a number of statistical methods to address this issue. Specifically, we (1) reported only the best-performing outcome per study group in the univariate meta-analysis, as this statistical analysis does not account for double sampling; (2) aggregated multiple outcomes reported on the same samples into one outcome for a bivariate meta-analysis to account for tests on the same cohort so as not to overestimate effect sizes; and (3) used three-level meta-analysis approach to explicitly model the hierarchical structure of the data, accounting for dependencies between outcomes nested within study groups and between-study group variance. Further longitudinal research in diverse clinical settings will be required to ensure findings can be generalised for clinical practice. Along with this, additional considerations of both the ethical boundaries and implementation approach for clinical services and their communities will be needed. Such efforts are critical for developing quantifiable, automated, streamlined detection and diagnosis tools to enhance clinical decision-making and provide objective markers for therapeutic response.

In conclusion, this study synthesises the existing literature testing AI-based methods for automated detection of social behaviour and diagnostic outcomes. The review highlights the significant potential for AI methods to inform and support clinical diagnoses, and to track social behaviour in real-world and laboratory environments. The potential for this technology to address current resource demands and to deliver objective outcomes may contribute to new frontiers for autism science.

## Contributors

Authors C.S., A.M., A.J.G., and E.A.D. conceptualised and designed the research study and registered the research protocol. C.S., A.K.S., and E.A.D. conducted literature searches and screening, and extracted data for analysis. C.S. and A.K.S. accessed and verified the underlying data. C.S. conducted the analysis with guidance from, and results verified by A.M., K.B., A.J.G., E.A.D., and A.L. All authors contributed and reviewed drafts of the manuscript and approved the final submission. All authors had full access to all the data in the study and had final responsibility for the decision to submit to publication.

## Data sharing statement

The data and customised R codes used to undertake this systematic review and meta-analysis are freely available at: https://github.com/CarterSunUSYD/Facial_AI_ASD_Diagnostic_MetaAnalysis.git. For any queries regarding access or use of the data and code, please contact the corresponding author at: adam.guastella@sydney.edu.au.

## Declaration of interests

We declare no competing interests.
