# Supplementary material for: Artificial intelligence for tracking social behaviours and supporting an autism spectrum disorder diagnosis: systematic review and meta-analysis
Source: eBioMedicine. 2025 Sep 26;120:105931. doi: 10.1016/j.ebiom.2025.105931 (PMC12509731; doi:10.1016/j.ebiom.2025.105931)
Supplement: Supplementary Tables and Figures [file mmc1.pdf]

Supplementary Materials

Supplementary Table S1. Literature search term.

Database	Final Search Terms
Embase	1. exp autism/<br>2. exp autism diagnostic observation schedule/<br>3. exp Autism Diagnostic Interview Revised/<br>4. exp autism assessment/<br>5. exp Asperger syndrome/<br>6. autis*.tw.<br>7. asperger*.tw.<br>8. ASD*.tw.<br>9. ADOS.tw.<br>10. 1 or 2 or 3 or 4 or 5 or 6 or 7 or 8 or 9<br>11. exp machine learning/<br>12. exp artificial intelligence/<br>13. exp algorithm/<br>14. exp artificial neural network/<br>15. exp automation/<br>16. exp deep learning/<br>17. exp computer vision/<br>18. exp image processing/<br>19. artificial intelligence.tw.<br>20. algorithm*.tw.<br>21. artificial neural network*.tw.<br>22. machine learning.tw.<br>23. deep neural network*.tw.<br>24. deep learning.tw.<br>25. exp computer vision/<br>26. automatic* detection*.tw.<br>27. automation.tw.<br>28. image process*.tw.<br>29. pattern recognition.tw.<br>30. 11 or 12 or 13 or 14 or 15 or 16 or 17 or 18 or 19 or 20 or 21 or 22 or 23 or 24 or 25 or 26 or 27 or 28 or 29<br>31. exp gaze/<br>32. exp eye fixation/<br>33. exp eye movement/<br>34. exp attention/<br>35. exp eye tracking/<br>36. eye gaz*.tw.<br>37. eye trac*.tw.<br>38. eye move*.tw.<br>39. eye fixation*.tw.<br>40. attention.tw.<br>41. eye contact.tw.<br>42. 31 or 32 or 33 or 34 or 35 or 36 or 37 or 38 or 39 or 40 or 41<br>43. exp emotion/<br>44. exp facial expression/<br>45. exp face/<br>46. exp facies/

	47. fac*.tw.
	48. emotion*.tw.
	49. 43 or 44 or 45 or 46 or 47 or 48
	50. exp social disability/
	51. exp developmental disorder/
	52. exp communication disorder/
	53. exp social behavior/
	54. socia*.tw.
	55. communication.tw.
	56. development* disorder.tw.
	57. exp play/
	58. exp play therapy/
	59. play*.tw.
	60. interact*.tw.
	61. 50 or 51 or 52 or 53 or 54 or 55 or 56 or 57 or 58 or 59 or 60
	62. 42 or 49
	63. 10 and 30 and 61 and 62
Medline	1. exp Autism Spectrum Disorder/
	2. exp Asperger syndrome/
	3. exp Autistic Disorder/
	4. Asperger*.tw.
	5. autis*.tw.
	6. ASD*.tw.
	7. ADOS.tw.
	8. 1 or 2 or 3 or 4 or 5 or 6 or 7
	9. exp Imaging, Three-Dimensional/
	10. exp Algorithms/
	11. exp Pattern Recognition, Automated/
	12. exp Artificial Intelligence/
	13. exp Image Enhancement/
	14. exp Image Processing, Computer-Assisted/
	15. exp Image Interpretation, Computer-Assisted/
	16. exp Deep Learning/
	17. exp Diagnosis, Computer-Assisted/
	18. exp Artificial Intelligence/
	19. exp Machine Learning/
	20. exp Computational Biology/
	21. exp Neural Networks, Computer/
	22. automatic* detection*.tw.
	23. deep learning.tw.
	24. machine learning*.tw.
	25. artificial neural network*.tw.
	26. deep neural network*.tw.
	27. artificial intelligence.tw.
	28. algorithm*.tw.
	29. pattern recognition.tw.
	30. image process*.tw.
	31. automation.tw.
	32. 9 or 10 or 11 or 12 or 13 or 14 or 15 or 16 or 17 or 18 or 19 or 20 or 21 or 22 or 23 or 24 or 25 or 26 or 27 or 28 or 29 or 30 or 31
	33. exp eye movements/
	34. exp fixation, ocular/

	35. exp Attention/<br>36. eye fixation*.tw.<br>37. eye gaz*.tw.<br>38. eye trac*.tw.<br>39. eye move*.tw.<br>40. attention.tw.<br>41. eye contact.tw.<br>42. 33 or 34 or 35 or 36 or 37 or 38 or 39 or 40 or 41<br>43. exp Facial Expression/<br>44. exp facies/<br>45. exp Emotions/<br>46. exp Facial Recognition/<br>47. fac*.tw.<br>48. emotion*.tw.<br>49. 43 or 44 or 45 or 46 or 47 or 48<br>50. exp Developmental Disabilities/<br>51. exp Social Behavior/<br>52. communication.tw.<br>53. socia*.tw.<br>54. 50 or 51 or 52 or 53<br>55. development* disorder.tw.<br>56. exp Play Therapy/<br>57. play*.tw.<br>58. exp Social Interaction/<br>59. interact*.tw.<br>60. 50 or 51 or 52 or 53 or 54 or 55 or 56 or 57 or 58 or 59<br>61. 42 or 49<br>62. 8 and 32 and 60 and 61
Scopus	((TITLE-ABS-KEY (autis*)) OR (TITLE-ABS-KEY (asd*)) OR (TITLE-ABS-KEY (asperge*)) OR (TITLE-ABS-KEY (ados))) AND ((TITLE-ABS-KEY (fac*)) OR (TITLE-ABS-KEY (emotion*)) OR (TITLE-ABS-KEY ("eye move*")) OR (TITLE-ABS-KEY (attention)) OR (TITLE-ABS-KEY ("eye trac*")) OR (TITLE-ABS-KEY ("eye gaz*")) OR (TITLE-ABS-KEY (eye AND fixation*)) OR (TITLE-ABS-KEY (eye AND contact))) AND ((TITLE-ABS-KEY ("socia*")) OR (TITLE-ABS-KEY ("social behavio*")) OR (TITLE-ABS-KEY ("social deplay*")) OR (TITLE-ABS-KEY ("social respons*")) OR (TITLE-ABS-KEY (communication)) OR (TITLE-ABS-KEY ("developmental disabil*")) OR (TITLE-ABS-KEY (play*)) OR (TITLE-ABS-KEY (interact*))) AND ((TITLE-ABS-KEY (algorithm*)) OR (TITLE-ABS-KEY ("Pattern Recognition")) OR (TITLE-ABS-KEY ("machine learning")) OR (TITLE-ABS-KEY ("artificial intelligence")) OR (TITLE-ABS-KEY ("artificial neural network")) OR (TITLE-ABS-KEY ("deep learning")) OR (TITLE-ABS-KEY ("deep neural network")) OR (TITLE-ABS-KEY ("automat* detect*")) OR (TITLE-ABS-KEY (auto*)) OR (TITLE-ABS-KEY ("image process*")))) AND (LIMIT-TO (DOCTYPE , "ar") OR LIMIT-TO (DOCTYPE , "cp"))
Web of Science Core Collection	(ALL=(autis* OR asperger* OR ASD OR ADOS))AND<br>(ALL=(fac* OR "eye gaze*" OR "eye contact" OR "eye trac*" OR "eye move*" OR "eye fixation*" OR emotion*)) AND<br>(ALL=("deep learning" OR "machine learning" OR algorithm* OR auto* OR "artific

	ial intelligence" OR "artificial neural network" OR "deep neural network" OR image process* OR pattern recognition)) AND<br>(ALL=("socia*" OR communication OR "development* disorder" OR play* OR inte ract*))<br>Indexes=SCI-EXPANDED, SSCI, A&HCI, CPCI-S, CPCI-SSH, ESCI, CCR-EXPANDED, IC Timespan=All years
ACM	AllField:(autis* OR asd* OR asperge* OR ADOS) AND AllField:(fac* OR emotion* OR "eye move*" OR "eye trac*" OR "eye gaz*" OR "eye fixation*" OR attention) AND AllField:(algorithm* OR "pattern recognition" OR "machine learning" OR "artificial intelligence" OR "artificial neural network" OR "deep learning" OR "deep neural network" OR "automat* detect*" OR "image process*" OR automation) AND AllField:("developmental disa*" OR communication OR "social delay*" OR "social respons*" OR "social behavio*" OR "socia*" OR play* OR interact*)
IEEE	(("All Metadata":autis* OR "All Metadata": asperge* OR "All Metadata": ASD OR "All Metadata": ADOS) AND ("All Metadata": fac* OR "All Metadata": emotion OR "All Metadata": "eye tracking" OR "All Metadata": "eye gaz*" OR "All Metadata": "eye movement" OR "All Metadata": attention OR "All Metadata": "eye fixation") AND ("All Metadata": "developmental disorder" OR "All Metadata": "socia*" OR "All Metadata": communication OR "All Metadata": play* OR "All Metadata": interact*) AND ("All Metadata": algorithm OR "All Metadata": "image processing" OR "All Metadata": "machine learning" OR "All Metadata": "artificial intelligence" OR "All Metadata": "pattern recognition" OR "All Metadata": "automatic detection" OR "All Metadata": automation OR "All Metadata": "deep learning" OR "All Metadata": "deep neural network" OR "All Metadata": "artificial neural network"))

Supplementary Table S2. Summary of the demographic characteristics of included studies.

Authors	Autism Diagnostic Tool	Type of Assessment	Autism Severity Level	Race/Ethnicity	Proportion of Male	IQ Selected Tool, IQ Composite score (Mean (SD))
A. Di Nuovo et al., 2018	ADI-R<br><br>(CARS-2, PEP-3, VABS)	Diagnosis	ASD (grade 3),<br>ID levels (4 mild to severe, 2 profound)	Predominantly White (European)/Italian	100%	Leiter-R, WISC,
C. Eunji et al., 2017	ADOS, ADI-R	Diagnostic	-	Caucasian 60 subjects, mixed 16 subjects, African American 10 subjects, Asian 6 subjects, Hispanic 4 subjects, unknown 4 subjects	74%	-
C. Tang et al., 2020	ADI-R, ADOS, DSM-5	Diagnostic	high-risk	Asian	81.9% (HR-ASD :43.37%, TD: 38.55%)	full-scale intelligence quotient
E. Chong et al., 2017	-	-	-	White/Caucasian (61% ASD, 60% TD), Black/African (4% ASD, 22% TD), American (Asian/Pacific Islander (15% ASD, 0% TD), More than one race (14% ASD, 13% TD), Other/unknown (6% ASD, 5% TD))	56% (TD: 62.5%, ASD:50%)	-

(Continued)

Supplementary Table S2. *(Continued)*

G. Nie et al., 2018	DSM-IV-TR<br><br>(ESCS test)	Diagnostic	-	Caucasian, African American, Asian	85.7% Dataset 1	-
H. Drimalla et al., 2019	ADOS and ADI-R<br><br>(AQ Questionnaire)	Diagnostic	-	-	51%	-
H. Drimalla et al., 2020	ADOS and ADI-R	Diagnostic	-	-	51%	Verbal Intelligence (Wortschatz test)
H. Javed et al., 2020	-	-	-	-	100%	-
K. S. Lohan et al., 2018	ADOS	Diagnostic	-	-	ASD:87.1%, TD: 87.9%	FSIQ-4 (the Weschler abbreviated scale of intelligence), (90.54 (12.21))
E. Chong et al., 2020	ADOS-2<br><br>(CSBS-DP, M-CHAT)	Diagnostic	-	White/Caucasian (61% ASD, 60% TD), Black/African (4% ASD, 22% TD), American (Asian/Pacific Islander (15% ASD, 0% TD), More than one race (14% ASD, 13% TD), Other/unknown (6% ASD, 5% TD))	ASD:83%, TD: 65%	-

(Continued)

Supplementary Table S2. *(Continued)*

Authors	Autism Diagnostic Tool	Type of Assessment	Autism Severity Level	Race/Ethnicity	Proportion of Male	IQ Selected Tool, IQ Composite score (Mean (SD))
D. Alie et al., 2011	ADOS, ADI-R, DSM-IV-TR criteria	Diagnostic	-	-	-	-
N. Qiu et al., 2020	M-CHAT, ADI-R and ADOS (2 years of age) (CSBS-DP, CARS and ABC)	Diagnosis	high-risk	Asian	ASD: 88.89%, TD: 74.42%	-
W. B. Liu et al., 2017	ADOS	Diagnostic	-	-	ASD: 72.73%, TD: 90.48%	-
N. Zhang et al., 2022	ADOS-2 Module 4 (CSS)	Diagnostic	-	-	ASD: 78.79%	Full-scale IQ (96.83 (13.48))
J. Zhang et al., 2022	ADI-R (ADOS)	Diagnostic	-	Asian	ASD: 88.1%, TD: 80.5%	-
M. Varma et al., 2022	-	-	-	-	ASD: 77.94%, TD: 66.67%	-
J. T. Megerian et al., 2022	clinicians	Diagnostic	-	American Indian, Asian, Black, Hawaiian or Pacific Islander, Non-Hispanic White, Hispanic or Latino, multiple races/ethnicities	ASD: 63.76%	-
I. Akin-Bulbul et al., 2022	DSM-5 (BAY-LEY-III, M-CHAT-R/F)	Diagnostic	-	-	-	-
M. Alcaniz et al., 2022	ADOS-2 module 1 (ADI-R intervein)	Diagnostic	-	Spanish	ASD: 66.66%, TD: 52.78%	-

(Continued)

Supplementary Table S2. *(Continued)*

Authors	Autism Diagnostic Tool	Type of Assessment	Autism Severity Level	Race/Ethnicity	Proportion of Male	IQ Selected Tool, IQ Composite score (Mean (SD))
A. Adham et al., 2022	-	-	-	-	ASD: 81.48%, TD: 46.88%	-
Z. Zhao et al., 2021	DSM-4	Diagnostic	-	Asian	ASD: 89.47%, TD: 85%	-
G. Zhang et al., 2021	Clinical psychology evaluation	Diagnosis	-	-	ASD: 70%	-
C. Wu et al., 2021	-	-	-	-	-	-
J. Li et al., 2021	ADOS-2	Diagnostic	-	-	-	-
P. R. Krishnappababu et al., 2021	ADOS-T (M-CHAT-R/F)	Diagnostic	-	White/Caucasian (77.7% TD, 47.5% TD), African American (15% ASD, 8.6% TD), Other/unknown (37.5% ASD, 13.6% TD))	ASD: 77.5%, TD: 48.9%	-
N. Kojovic et al., 2021	DSM-5, ADOS-G, ADOS-2	Diagnostic	-	-	ASD: 58.82% + 99%; TD: 58.82%	-
D. Anagnostopoulou et al., 2021	-	-	-	-	ASD: 71.43%	-
C. Ko et al., 2023	K-CARS-2, K-ADOS-2	Diagnostic	-	Korean	ASD: 53.3%, TD: 54%	Korean Bayley Scales of Infant and Toddler Development, Second Edition; Korean Wechsler Preschool and Primary Scale of Intelligence, Fourth Edition

(Continued)

Supplementary Table S2. *(Continued)*

Authors	Autism Diagnostic Tool	Type of Assessment	Autism Severity Level	Race/Ethnicity	Proportion of Male	IQ Selected Tool, IQ Composite score (Mean (SD))
V. G. Prakash et al., 2023	Not provided	Diagnostic	-	Indian	-	
P. R. Krishnappababu et al., 2023a	M-CHAT-R/F, ADOS-2	Diagnostic	-	American Indian/Alaskan Native; Asian; Black or African American; White/Caucasian; More than one race; Other	ASD: 70.73%, TD: 50.24%	-
P. R. Krishnappababu et al., 2023b	M-CHAT-R/F, ADOS-2	Diagnostic	-	American Indian/Alaskan Native; Asian; Black or African American; White/Caucasian; More than one race; Other	ASD: 74.42%, TD: 50.24%	-
J. Liu et al., 2023	Mentioned clinicians only	Diagnostic	-	Asian	-	-
M. E. Minissi et al., 2024	ADOS-2	Diagnostic	-	Spanish	ASD: 82.05%, TD: 45.24%	-
W. Saakyan et al., 2023	ICD-10	Diagnostic	-	-	ASD: 57.83%, TD: 49.38%	-
D. Q. McDonald et al., 2023	DSM-5	Diagnostic	-	-	85.71%	-
Z. Zhao et al., 2024	-	-	-	Korean	-	-
X. Wang et al., 2024	ADI-R and ADOS	Diagnostic	-	Asian	70.83%	-
L. L. Liu et al., 2024	ABC (Behavioural Rating Scale)	Screening	-	Asian	ASD: 68.97%, TD: 66.67%	-

Supplementary Table S3. Summary of the experimental tasks of the included studies.

Study	Type of Experiment	Experiment Duration Description
A. Di Nuovo et al., 2018	Therapy	14 encounters over one month, 3 sessions per week (6-8 min per child/session)
C. Eunji et al., 2017	Assessment +Therapy	Dataset 1: v-BOSCC (12-minute examiner-child interaction (2x5-minutes play segments with standardized sets of toys, and a 2-minute conversation segment)); Dataset2: ESCS (15-25 minute structured assessment uses standardized toys) and v-BOSCC (2x4-minutes play and 2-minute snake); Dataset 3: Rapid-ABC assessment (R-ABC), 2-to-4 minute play (5 activities); Dataset 4: Marcus therapy [1) pairing, 12 sessions; 2)Demands, 5 sessions; 3) Mands, 6 sessions], 10-minute each session, except two sessions (3 and 9 minutes)
C. Tang et al., 2020	Assessment	the mother amused baby without a touch of the body (2 minutes) + 1-minute still-face episode.
E. Chong et al., 2017	Assessment	ESCS (15-25 minutes)
H. Drimalla et al., 2019	Screening	Simulated Interaction Task (7minutes)
H. Drimalla et al., 2020	Screening	Simulated Interaction Task (7minutes)
H. Javed et al., 2020	Intervention	8-10 minutes per robot x2
K. S. Lohan et al., 2018	Intervention	-
E. Chong et al., 2020	Assessment	ESCS (15-25 minutes), BOSCC (10-12 minutes)
D. Alie et al., 2011	Intervention	5 minutes
N. Qiu et al., 2020	Screening	-
J. Zhang et al., 2022	Assessment	12 clips (5-7 seconds/clip)
M. Varma et al., 2022	Assessment	90 seconds
M. Alcaniz et al., 2022	Assessment	24 minutes and 45 seconds
I. Akin-Bulbul et al., 2022	Screening	48 seconds for the stimulus (16x3), 2-second fixation before each stimulus
Z. Zhao et al., 2021	Screening	-

(Continued)

Supplementary Table S3. *(Continued)*

Study	Type of Experiment	Experiment Duration Description
P. R. Krishppababu et al., 2021	well-child visit (could be both screening/assessment)	about 5 minutes
D. Anagnostopoulou et al., 2021	Therapy	20 minutes
J. Li et al., 2021	Therapy	-
C. Wu et al., 2021	Screening	3 minutes
G. Zhang. et al., 2021	Therapy	15 minutes
N. Zhang et al., 2022	Assessment	1 hour
J. T. Megerian et al., 2022	Screening	5 minutes
C. Ko et al., 2023 [57]	Assessment	10 minutes
V. G. Prakash et al., 2023	Therapy	20 minutes
P. R. Krishnappababu et al., 2023a	Assessment	-
P. R. Krishnappababu et al., 2023b	Assessment	8 minutes
J. Liu et al., 2023	Assessment	5-10 minutes
M. E. Minissi et al., 2024	Assessment	-
W. Saakyan et al., 2023	Assessment	7 minutes
D. Q. McDonald et al., 2023	Assessment	3 minutes
Z. Zhao et al., 2024	Assessment	30 minutes
X. Wang et al., 2024	Assessment	-
L. L. Liu et al., 2024	Assessment	-

Supplementary Table S4. Summary of the model training details and performance metrics reported in the included studies.

Study	Input Feature Type	Algorithms	Performance
A. Di Nuovo et al., 2018	Face Detection	Naïve Classification	Acc:0.734, Precision: 0.706, Sensitivity: 0.678, Specificity: 0.776, NPV: 0.696, AUC: 0.738, F1: 0.692
		K-NN	Acc:0.754, Precision:0.729, Sensitivity:0.651, Specificity:0.812, NPV: 0.706, AUC: 0.793, F1:0.688
		Naïve Classification	Acc: 0.696, Precision: 0.663, Sensitivity: 0.759, Specificity: 0.642, NPV: 0.583, AUC: 0.868, F1: 0.708
		K-NN	Acc: 0.846, Precision: 0.745, Sensitivity: 0.858, Specificity: 0.767, NPV: 0.782, AUC: 0.931, F1: 0.798
			Acc: 0.709, Precision: 0.619, Sensitivity: 0.953, Specificity: 0.483, NPV: 0.902, AUC: 0.619, F1: 0.751
			Acc: 0.882, Precision: 0.833, Sensitivity: 0.830, Specificity: 0.873, NPV: 0.818, AUC: 0.913, F1: 0.831
C. Eunji et al., 2017	Eye Contact Detection	PiCNN	Precision: 0.750, Sensitivity: 0.800, AUC: 0.790, F1: 0.780, Matthews correlation coefficient: 0.770
		AlexNet	Precision: 0.710, Sensitivity: 0.770, AUC: 0.750, F1: 0.730, Matthews correlation coefficient: 0.720
		PEEC	Acc: 0.670, Precision: 0.590, AUC: 0.570, F1: 0.630, Matthews correlation coefficient: 0.620

(Continued)

Supplementary Table S4. *(Continued)*

Study	Input Feature Type	Algorithms	Performance
C. Tang et al., 2020	Head Movement Features	SVM	Acc: 0.590, Precision: 0.546, Sensitivity: 0.900, Specificity: 0.302, AUC: 0.577, F1:0.679
			Acc: 0.892, Precision: 0.919, Sensitivity: 0.850, Specificity: 0.930, AUC: 0.892, F1: 0.883
			Acc: 0.880, Precision: 0.857, Sensitivity: 0.900, Specificity: 0.861, AUC: 0.848, F1: 0.878
			Acc: 0.868, Precision: 0.837, Sensitivity: 0.900, Specificity: 0.837, AUC: 0.831, F1: 0.867
			Acc: 0.904, Precision: 0.944, Sensitivity: 0.850, Specificity: 0.954, AUC: 0.898, F1: 0.895
			Acc: 0.964, Precision: 0.974, Sensitivity: 0.950, Specificity: 0.977, AUC: 0.946, F1: 0.962
			Acc: 0.964, Precision: 0.974, Sensitivity: 0.950, Specificity: 0.977, AUC: 0.946, F1: 0.962
		K-NN	Acc: 0.542, Precision: 0.531, Sensitivity: 0.425, Specificity: 0.651, AUC: 0.541, F1: 0.472
			Acc: 0.807, Precision: 0.929, Sensitivity: 0.650, Specificity: 0.953, AUC: 0.779, F1: 0.765
			Acc: 0.868, Precision: 0.837, Sensitivity: 0.900, Specificity: 0.837, AUC: 0.852, F1: 0.867
			Acc: 0.880, Precision: 0.857, Sensitivity: 0.900, Specificity: 0.861, AUC: 0.858, F1: 0.878
			Acc: 0.807, Precision: 0.929, Sensitivity: 0.650, Specificity: 0.954, AUC: 0.779, F1: 0.765
			Acc: 0.940, Precision: 0.949, Sensitivity: 0.925, Specificity: 0.954, AUC: 0.925, F1:0.9367
			Acc: 0.928, Precision: 0.947, Sensitivity: 0.900, Specificity: 0.954, AUC: 0.914, F1: 0.923
E. Chong et al., 2017	Gaze Shift for Initiating Joint Attention	SVM	AUC: 0.780<br>AUC: 0.800

(Continued)

Supplementary Table S4. *(Continued)*

Study	Input Feature Type	Algorithms	Performance
H. Drimalla et al., 2019	Facial expression, gaze, audio	Random Forest	Acc: 0.753, Precision: 0.774, Sensitivity: 0.667, Specificity: 0.829, AUC: 0.840, F1: 0.716
		SVM	Acc: 0.714, Precision: 0.719, Sensitivity: 0.639, Specificity: 0.780, AUC: 0.810, F1: 0.676
		Stacked CNN	Acc: 0.461, Precision: 0.391, Sensitivity: 0.250, Specificity: 0.650, AUC: 0.530, F1: 0.305
		Pooled CNN	Acc: 0.605, Precision: 0.594, Sensitivity: 0.528, Specificity: 0.675, AUC: 0.640, F1: 0.559
H. Drimalla et al., 2020	Facial AU features	Random Forest	Acc: 0.671, Precision: 0.647, Sensitivity: 0.611, Specificity: 0.721, AUC: 0.740, F1: 0.629
			Acc: 0.570, Precision: 0.528, Sensitivity: 0.528, Specificity: 0.605, AUC: 0.630, F1: 0.528
			Acc: 0.747, Precision: 0.722, Sensitivity: 0.722, Specificity: 0.767, AUC: 0.770, F1: 0.722
			Acc: 0.730, Precision: 0.727, Sensitivity: 0.667, Specificity: 0.791, AUC: 0.780, F1: 0.696
K. S. Lohan et al., 2018	Pupil Diameter Data	LSTM	Acc: 0.798
	AOI		Acc: 0.759
E. Chong et al., 2020	Cropped Face Regions	Deep model (smoothed) (ResNet-50 backbone)	Precision: 0.936, Sensitivity: 0.943, F1: 0.940<br>Precision: 0.924, Sensitivity: 0.937, F1: 0.930
		Deep model (without transfer learning smoothed)	Precision: 0.917, Sensitivity: 0.915, F1: 0.916
D. Alie et al., 2011	Gaze Pattern	VMM	Acc: 0.938, Precision: 0.750, Sensitivity: 1, Specificity: 0.923, F1: 0.857
		HMM	Acc: 0.813, Precision: 0.5, Sensitivity: 0.333, Specificity: 0.923, F1:0.400

(Continued)

Supplementary Table S4. *(Continued)*

Study	Input Feature Type	Algorithms	Performance
J. Zhang et al., 2022	valid sampling rate, number of RJA events	Logistic regression	Acc: 0.760, AUC: 0.818
M. Varma et al., 2022	Gaze fixation pattern	LSTM (window= 100, shift = 10)	Precision: 0.595, Sensitivity: 0.598
M. Alcaniz et al., 2022	(9 features) Number of AOIs seen, average number of AOIs seen per scene (and SD), number of frames with no AOIs, AOIs, persons, items	SVM	Acc: 0.860, Sensitivity: 0.910, Specificity: 0.820, AUC: 0.910, F1: 0.870
			Acc: 0.770, Sensitivity: 0.780, Specificity: 0.850, AUC: 0.830, F1: 0.790
			Acc: 0.840, Sensitivity: 0.940, Specificity: 0.710, AUC: 0.880, F1: 0.870
		Random Forest	Acc: 0.820, Sensitivity: 0.920, Specificity: 0.640, AUC: 0.840, F1: 0.860
		Naïve Bayes	Acc: 0.830, Sensitivity: 0.820, Specificity: 0.850, AUC: 0.840, F1: 0.840
		SVM	Acc: 0.780, Sensitivity: 0.900, Specificity: 0.610, AUC: 0.790, F1: 0.820
		XGBoost	Acc: 0.730, Sensitivity: 0.800, Specificity: 0.680, AUC: 0.770, F1: 0.770
		K-NN	Acc: 0.740, Sensitivity: 0.880, Specificity: 0.540, AUC: 0.670, F1: 0.790
			Acc: 0.740, Sensitivity: 0.880, Specificity: 0.540, AUC: 0.670, F1: 0.790
			Acc: 0.660, Sensitivity: 0.730, Specificity: 0.510, AUC: 0.640, F1: 0.700
		Naïve Bayes	Acc: 0.690, Sensitivity: 0.670, Specificity: 0.710, AUC: 0.720, F1: 0.710

(Continued)

Supplementary Table S4. *(Continued)*

Study	Input Feature Type	Algorithms	Performance
Z. Zhao et al., 2021	Gaze fixation time (three features)	SVM	Acc: 0.923, Precision: 1, Sensitivity: 0.842, Specificity: 1, AUC: 0.920, F1: 0.914
		LDA	Acc: 0.897, Sensitivity: 0.895, Specificity: 0.900, AUC: 0.920
		Decision Tree	Acc: 0.846, Sensitivity: 0.895, Specificity: 0.800, AUC: 0.860
		Random Forest	Acc: 0.846, Sensitivity: 0.842, Specificity: 0.850, AUC: 0.860
P. R. Krishppababu et al., 2021	Integrated entropy	Decision Tree	Acc: 0.783, Precision: 0.724, Sensitivity: 0.935, F1: 0.816
			Acc: 0.783, Precision: 0.705, Sensitivity: 0.888, F1: 0.786
			Acc: 0.766, Precision: 0.700, Sensitivity: 0.893, F1: 0.785
			Acc: 0.767, Precision: 0.703, Sensitivity: 0.896, F1: 0.788
			Acc: 0.750, Precision: 0.750, Sensitivity: 0.770, F1: 0.760
			Acc: 0.650, Precision: 0.751, Sensitivity: 0.825, F1: 0.786
			Acc: 0.700, Precision: 0.691, Sensitivity: 0.821, F1: 0.750
			Acc: 0.616, Precision: 0.625, Sensitivity: 0.517, F1: 0.566
			Acc: 0.791, Precision: 0.757, Sensitivity: 0.774, F1: 0.765
			Acc: 0.795, Precision: 0.751, Sensitivity: 0.851, F1: 0.798
			Acc: 0.750, Precision: 0.724, Sensitivity: 0.750, F1: 0.737
			Acc: 0.633, Precision: 0.613, Sensitivity: 0.655, F1: 0.633
N. Kojovic et al., 2021	Movement and 5seconds of video	VGG16 CNN + LSTM	Acc: 0.809, Precision: 0.784, Sensitivity: 0.854, Specificity: 0.765, F1: 0.818

(Continued)

Supplementary Table S4. *(Continued)*

Study	Input Feature Type	Algorithms	Performance
A. Adham et al., 2022	Eye Gaze scan path (Spatial)	DNN	Acc: 0.744, AUC: 0.6828
		CNN	Acc: 0.725, AUC: 0.721
			Acc: 0.763, AUC: 0.818
			Acc: 0.803, AUC: 0.839
W. B. Liu et al., 2017	Latency	Decision Tree	Acc: 0.907
			Acc: 0.907
			Acc: 0.930
J. Li et al., 2021	log-Mel spectrogram + Image	ResNet-18	Acc: 0.724, F1: 0.750
C. Wu et al., 2021	Image, Facial Key points Features	ResNet-18	Acc: 0.645, F1: 0.645
G. Zhang et al., 2021	social visual behaviour scores	Deep CNN (Three branches)	Mean Squared error loss:0.177
N. Zhang et al., 2022	LPQ-TOP scenes	SVM	Acc:0.917, F1:0.901
J. T. Megerian et al., 2022	video and questionnaires	gradient boosted decision tree	Sensitivity:0.984, Precision: 0.789

(Continued)

Supplementary Table S4. *(Continued)*

Study	Input Feature Type	Algorithms	Performance
D. Anagnostopoulou et al., 2021	Pose features (TD-Joint Attention Data set)	1D CNN	Acc: 0.730, Precision: 0.563, F1: 0.602
		ResNet-50	Acc: 0.745, Precision: 0.633, F1: 0.645
		LSTM (one layer)	Acc: 0.762, Precision: 0.744, F1: 0.742
		1D CNN	Acc: 0.774, Precision: 0.763, F1: 0.752
		2D CNN	Acc: 0.789, Precision: 0.774, F1: 0.765
		LSTM	Acc: 0.795, Precision: 0.780, F1: 0.769
		AlexNet	Acc: 0.804, Precision: 0.807, F1: 0.805
		1D CNN	Acc: 0.679, Precision: 0.681, F1: 0.680
		2D CNN	Acc: 0.709, Precision: 0.736, F1: 0.713
		LSTM	Acc: 0.718, Precision: 0.715, F1: 0.716
		AlexNet	Acc: 0.787, Precision: 0.824, F1: 0.734
		2D CNN	Acc: 0.758, Precision: 0.770, F1: 0.754
		2D CNN	Acc: 0.709, Precision: 0.736, F1: 0.713
		LSTM	Acc: 0.615, Precision: 0.621, F1: 0.605
		LSTM	Acc: 0.718, Precision: 0.715, F1: 0.716
		AlexNet	Acc: 0.672, Precision: 0.678, F1: 0.643
		AlexNet	Acc: 0.787, Precision: 0.824, F1: 0.734

(Continued)

Supplementary Table S4. *(Continued)*

Study	Input Feature Type	Algorithms	Performance
I. Akin-Bulbul et al., 2022	Fixation count, Fixation duration, Dwell time, Net Dwell Time, Diversion Duration (Relief feature selection)	Decision Tree	Acc: 0.725, AUC: 0.736, F1: 0.721
		Naïve Bayes	Acc: 0.825, AUC: 0.843, F1: 0.825
		Random Forest	Acc: 0.720, AUC: 0.759, F1: 0.716
		SVM	Acc: 0.775, AUC: 0.775, F1: 0.768
	Fixation count, Fixation duration, Dwell time, Net Dwell Time, Diversion Duration (InfoGain feature selection)	Decision Tree	Acc: 0.725, AUC: 0.736, F1: 0.721
		Naïve Bayes	Acc: 0.850, AUC: 0.845, F1: 0.848
		Random Forest	Acc: 0.725, AUC: 0.825, F1: 0.71
		SVM	Acc: 0.825, AUC: 0.825, F1: 0.822
	Fixation count, Fixation duration, Dwell time, Net Dwell Time, Diversion Duration (Wrapper feature selection)	Decision Tree	Acc: 0.725, AUC: 0.659, F1: 0.710
		Naïve Bayes	Acc: 0.800, AUC: 0.835, F1: 0.836
		Random Forest	Acc: 0.800, AUC: 0.794, F1: 0.795
		SVM	Acc: 0.750, AUC: 0.750, F1: 0.744

(Continued)

Supplementary Table S4. *(Continued)*

Study	Input Feature Type	Algorithms	Performance
I. Akin-Bulbul et al., 2022	Fixation count, Fixation duration, Dwell time, Net Dwell Time, Diversion Duration (Relief feature selection)	Decision Tree	Acc: 0.800, AUC: 0.800, F1: 0.792
		Naïve Bayes	Acc: 0.825, AUC: 0.820, F1: 0.825
		Random Forest	Acc: 0.875, AUC: 0.888, F1: 0.874
		SVM	Acc: 0.875, AUC: 0.875, F1: 0.874
	Fixation count, Fixation duration, Dwell time, Net Dwell Time, Diversion Duration (InfoGain feature selection)	Decision Tree	Acc: 0.675, AUC: 0.678, F1: 0.675
		Naïve Bayes	Acc: 0.800, AUC: 0.815, F1: 0.815
		Random Forest	Acc: 0.85, AUC: 0.874, F1: 0.848
		SVM	Acc: 0.800, AUC: 0.800, F1: 0.798
	Fixation count, Fixation duration, Dwell time, Net Dwell Time, Diversion Duration (Wrapper feature selection)	Decision Tree	Acc: 0.825, AUC: 0.825, F1: 0.819
		Naïve Bayes	Acc: 0.825, AUC: 0.858, F1: 0.824
		Random Forest	Acc: 0.800, AUC: 0.823, F1: 0.795
		SVM	Acc: 0.800, AUC: 0.800, F1: 0.795

(Continued)

Supplementary Table S4. *(Continued)*

Study	Input Feature Type	Algorithms	Performance
H. Javed et al., 2020	Movement + Expression	CNN (TD)	Acc: 0.680, F1: 0.650
		SVC (TD)	Acc: 0.640, F1: 0.580
		Random Forest (TD)	Acc: 0.740, F1: 0.740
		Decision Tree (TD)	Acc: 0.640, F1: 0.610
		K-NN (TD)	Acc: 0.740, F1: 0.730
		CNN (ASD)	Acc: 0.720, F1: 0.710
		SVC (ASD)	Acc: 0.600, F1: 0.580
		Random Forest (ASD)	Acc: 0.770, F1: 0.760
		Decision Tree (ASD)	Acc: 0.610, F1: 0.600
		K-NN (ASD)	Acc: 0.760, F1: 0.760
		CNN (ASD+ TD)	Acc: 0.650, F1: 0.620
		SVC (ASD + TD)	Acc: 0.590, F1: 0.540
		Random Forest (ASD + TD)	Acc: 0.740, F1: 0.710
		Decision Tree (ASD + TD)	Acc: 0.600, F1: 0.560
		K-NN (ASD + TD)	Acc: 0.710, F1: 0.710

(Continued)

Supplementary Table S4. *(Continued)*

Study	Input Feature Type	Algorithms	Performance
N. Qiu et al., 2020	behavioural characteristics	Naïve Bayes (FF episode)	Acc: 0.805, Precision: 0.811, Sensitivity: 0.750, Specificity: 0.837, F1: 0.779
		Naïve Bayes (SF episode)	Acc: 0.824, Precision: 0.838, Sensitivity: 0.775, Specificity: 0.860, F1: 0.805
		Random Forest (FF episode)	Acc: 0.807, Precision: 0.816, Sensitivity: 0.775, Specificity: 0.837, F1: 0.795
		Random Forest (SF episode)	Acc: 0.831, Precision: 0.861, Sensitivity: 0.775, Specificity: 0.884, F1: 0.816
		SVM (FF episode)	Acc: 0.812, Precision: 0.778, Sensitivity: 0.875, Specificity: 0.767, F1: 0.824
		SVM (SF episode)	Acc: 0.834, Precision: 0.829, Sensitivity: 0.850, Specificity: 0.837, F1: 0.840
C. Ko et al., 2023	Video recordings of joint attention moments	ResNet18-LSTM-Attention (Initiation of Joint Attention episode)	Acc: 0.976, AUC: 0.996, Precision: 0.955, Sensitivity: 0.992
		ResNet18-LSTM-Attention (Low-level Response to Joint Attention)	Acc: 0.784, AUC: 0.844, Precision: 0.747, Sensitivity: 0.784
		ResNet18-LSTM-Attention (High-level Response to Joint Attention)	Acc: 0.810, AUC: 0.842, Precision: 0.686, Sensitivity: 0.81
Krishnappababu et al., 2023a	Head Movements	SVM	Feature: the absolute mean of the acceleration; AUC: 0.85 [0.75,0.94]<br>Feature: Integrated Entropy; AUC: 0.8 [0.70, 0.89]<br>Feature: the absolute mean of the acceleration and integrated entropy; AUD: 0.73 [0.62, 0.84]

(Continued)

Supplementary Table S4. *(Continued)*

Study	Input Feature Type	Algorithms	Performance
Krishnappa Babu, P. R. et al., 2023b	total time facing forward	Linear Logistic Regression	AUC:0.73
	total time facing forward + mean blink rate during social task		AUC: 0.77
	percentage of time the child gazed at the social elements		AUC:0.76
	mean blink rate + percentage of time gaze at the social element		AUC: 0.75
	Fusion of all above features		AUC:0.82
J. Liu et al., 2023	Facial and Skeleton Feature from Images	CNN +Spatial-Temporal Graph Convolutional Network	Acc: 0.794, Sensitivity: 0.703, F1:0.71
	Facial and Skeleton Feature from Video		Acc: 0.854, Sensitivity: 0.833, F1:0.826
M. E. Minissi et al., 2024	posture movement,	Linear SVM	Acc: 0.81, Sensitivity: 0.72, Specificity:0.89, AUC:0.89
	gaze		Acc: 0.59, Sensitivity: 0.29, Specificity:0.82, AUC:0.89
	response to tasks		Acc: 0.75, Sensitivity: 0.57, Specificity:0.91, AUC:0.8
W. Saakyan et al., 2023	Facial Expression features	XGBoost	Acc: 0.73, Precision: 0.73, Sensitivity:0.75, AUC:0.81
	Voice Features		Acc: 0.7, Precision: 0.71, Sensitivity:0.66, AUC:0.78
	Gaze		Acc: 0.55, Precision: 0.56, Sensitivity:0.55, AUC:0.63
	Head movement features		Acc: 0.68, Precision: 0.68, Sensitivity:0.7, AUC:0.77
	Early Fusion (Facial Expression,Voice, Gaze, Head Movement)		Acc: 0.66, Precision: 0.68, Sensitivity:0.63, AUC:0.77
	Late Fusion (Facial Expression,Voice, Gaze, Head Movement)		Acc: 0.74, Precision: 0.73, Sensitivity:0.76, AUC:0.84

(Continued)

Supplementary Table S4. *(Continued)*

Study	Input Feature Type	Algorithms	Performance
D. Q. McDonald et al.,2023	Head movement features (monadic)	SVM	Acc: 0.692, Precision: 0.575, Recall: 0.545, Specificity: 0.774, NPV: 0.755
	Head movement features (Dyadic)		Acc: 0.8, Precision: 0.824, Recall: 0.559, Specificity: 0.933, NPV: 0.792
	Head movement features (monadic + Dyadic)		Acc: 0.797, Precision: 0.8, Recall: 0.56, Specificity: 0.926, NPV: 0.781
Z. Zhao et al., 2024	Image	CNN (SlowFast+BERT)	Acc: 0.74, Precision: 0.33, Recall: 0.69, AUC: 0.4
X. Wang et al., 2024	Facial	Feature Clustering Network + Multi-head Attention Network + Attention Fusion Network	Acc: 0.9167, Precision: 0.9231, Recall: 0.9231, F1: 0.923
	Posture	MMPose +Yolo	Acc: 0.9167, Precision: 1, Recall: 0.8667, F1: 0.929
	Posture	MMPose +Yolo	Acc: 0.9167, Precision: 0.9048, Recall: 0.9487, F1: 0.917
	Gaze	Restnet-18	Acc: 1, Precision: 1, Recall: 1, F1: 1
	Gaze	Restnet-18	Acc: 0.9167, Precision: 1, Recall: 0.8824, F1: 0.938
L. L. Liu et al., 2024	Gaze	TCN (Temporal Convolutional Networks)	Acc: 0.7458, Recall: 0.8275, Specificity: 0.6667, AUC: 0.7213
		Bi-LSTM	Acc: 0.8475, Recall: 0.9655, Specificity: 0.7333, AUC: 0.8897
		GRUA (Gated Recurrent Unit with Attention)	Acc: 0.7797, Recall: 0.6552, Specificity: 0.9, AUC:0.8276
		GNM (GoogLeNet with SVM)	Acc: 0.8333, Recall: 0.6667, Specificity: 1, AUC: 0.875
		TDEA-Net	Acc: 0.9492, Recall: 0.931, Specificity: 0.9667, AUC: 0.9517

Supplementary Table S5. Summary of feature extraction algorithms and tools used in the included studies.

Authors	Facial and Head Pose Estimation	Movement Feature	Gaze Estimation/ Eye Contact Algorithms	Vocal Features Extraction
A. Di Nuovo et al., 2018	Viola-Jones, MTCNN, Faster R-CNN	-	-	-
C. Eunji et al., 2017	Faster-RCNN	-	PEEC Detector (Pose-Dependent Egocentric Eye Contact Detector), PiCNN Detector (Pose-implicit Convolutional Neural Networks), Modified AlexNet, Gaze Locking	-
C. Tang et al., 2020	MTCNN, HOG	-	-	openSMILE (low-level descriptors)
E. Chong et al., 2017	Omron OKAO Library, IntraFace (facial landmarks) + Perspective-n-Point Algorithm (head pose)	-	-	-
G. Nie et al., 2018	Gaussian Mixture Model, hard histogram of head pose, soft histogram of head pose	hard histogram of head pose motion, soft histogram of head pose motion	-	-
H. Drimalla et al., 2019	OpenFace, HOG	-	-	librosa library, proscodic (root-mean-square energy), spectral features (forty mel-frequency cepstral coefficients, MMCC)
H. Drimalla et al., 2020	OpenFace	-	-	-
H. Javed et al., 2020	OpenPose	OpenPose, Laban Movement Analysis	-	-
K. S. Lohan et al., 2018	-	-	Pupil diameter and AOI	-
E. Chong et al., 2020	-	-	-	-
D. Alie et al., 2011	-	-	-	-

(Continued)

Supplementary Table S5. *(Continued)*

Authors	Facial and Head Pose Estimation	Movement Feature	Gaze Estimation/ Eye Contact Algorithms	Vocal Features Extraction
N. Qiu et al., 2020	-	-	Duration and frequency of eye contact, active social engagement, and social smiling during the Face-to-face episode and still-face episode	-
W. B. Liu et al., 2017	Dlib, landmarks (head pose) + PCA	-	-	Kaldi
N. Zhang et al., 2022	MTCNN, local phase quantization in three orthogonal planes (LPQ-TOP), histogram	-	-	-
J. Zhang et al., 2022	-	-	Eye Tracker	-
M. Varma et al., 2022	Amazon Rekognition, OpenFace	-	OpenFace, Coarse gaze annotation (16 predefined AOIs)	-
J. T. Megerian et al., 2022	-	-	-	-
M. Alcaniz et al., 2022	-	-	-	-
I. Akin-Bulbul et al., 2022	-	-	-	-
A. Adham et al., 2022	-	-	-	-
Z. Zhao et al., 2021	-	-	Tobii	-
G. Zhang et al., 2021	-	-	-	-
C. Wu et al., 2021	OpenFace 2.0	-	-	-
J. Li et al., 2021	MTCNN	-	-	log-Mel spectrograms
P. R. Krishnappababu et al., 2021	low-dimensional facial embedding	-	-	-
N. Kojovic et al., 2021	-	OpenPose	-	-
D. Anagnostopoulou et al., 2021	-	OpenPose	-	-
C. Ko et al., 2023	-	-	-	-
V. G. Prakash et al., 2023	Faster-RCNN and Resnet-50, Resnet-34 based facial expression recognition model	YOLO-V3, Euler angles	-	-
P. R. Krishnappababu et al., 2023a	Dlib-ml, OpenFace2.0	OpenFace2.0	iTracker and Otsu Method	-

(Continued)

Supplementary Table S5. *(Continued)*

Authors	Facial and Head Pose Estimation	Movement Feature	Gaze Estimation/ Eye Contact Algorithms	Vocal Features Extraction
Krishnappa Babu, P. R. et al., 2023b	Dlib-ml, OpenFace2.0	OpenFace2.0	iTracker and Otsu Method	-
J. Liu et al., 2023	FaceX-Zoo	AlphaPose		-
M. E. Minissi et al., 2024		Azure Kinect DK	Tobii Pro Glasses 2, Velocity threshold algorithm	-
W. Saakyan et al., 2023	OpenFace 2.2	-	OpenFace 2.2, gaze movement velocity and acceleration, saccade amplitude, fixation duration	-
D. Q. McDonald et al.,2023	3D facial analysis algorithm	K-means	-	-
Z. Zhao et al., 2024	-	-	-	-
X. Wang et al., 2024	Feature Clustering Network + Multi-head Attention Network + Attention Fusion Network	Restnet-18, MMPose	-	-
L. L. Liu et al., 2024	-	-	-	-

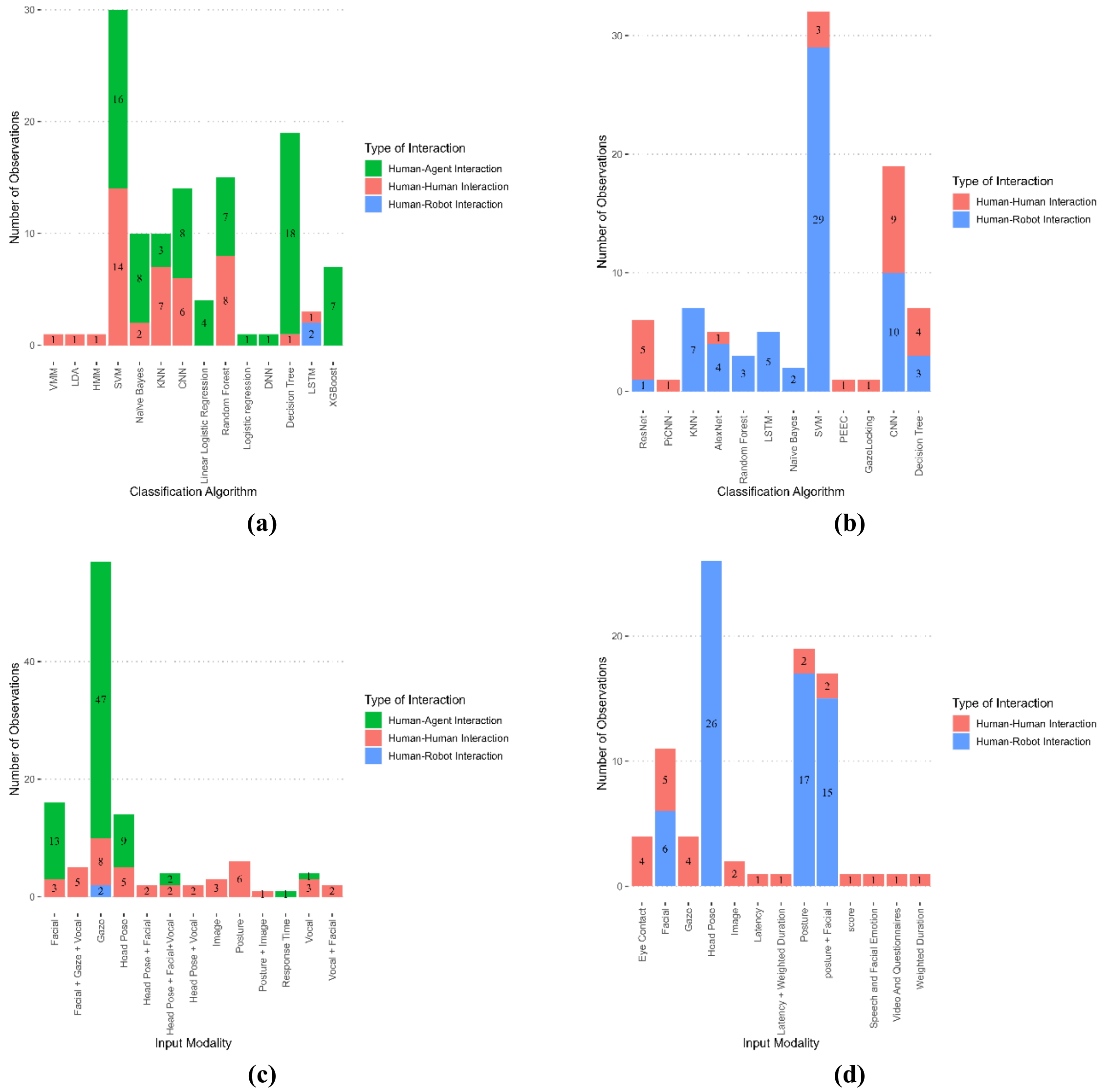

Supplementary Figure S1. Bar chart illustrating the input modalities and the classification algorithms applied in autism diagnosis and social behaviour detection studies. For all plots, the results are displayed in descending order of accuracy. The subplots are categorised based on the type of social interactions: Human-to-Agent interaction (green), Human-to-Human interaction (red) and Human-to-Robot interaction (blue). Subfigure a) presents the number of observations (outcomes) corresponding to different classification algorithms employed in autism diagnosis. Subfigure b) presents the use of the classification algorithm in behaviour detection studies. Subfigure c) presents the types of input modalities used for training AI models in autism diagnosis studies. Subfigure d) presents the types of input modalities used in behaviour detection studies.

Supplementary Table S6. Contingency table for the meta-analysis.

Study	Sample Size (ASD/TD)	Experimental Tasks	Classification Algorithm	Modality	TP	FP	TN	FN	Sensitivity	Specificity
C. Tang et al., 2020	40/43	Still-Face	SVM	Head Pose	36	30	13	4	0.9	0.302
				Facial	34	3	40	6	0.85	0.930
				Vocal	36	6	37	4	0.9	0.861
				Head Pose + Vocal	36	7	36	4	0.9	0.837
				Head Pose + Facial	34	2	41	6	0.85	0.954
				Vocal + Facial	38	1	42	2	0.95	0.977
				Head Pose+ Facial+ Vocal	38	1	42	2	0.95	0.977
			K-NN	Head Pose	17	15	28	23	0.425	0.651
				Facial	26	2	41	14	0.65	0.953
				Vocal	36	7	36	4	0.9	0.837
				Head Pose + Vocal	36	6	37	4	0.9	0.861
				Head Pose + Facial	26	2	41	14	0.65	0.954
				Vocal + Facial	37	2	41	3	0.925	0.954
				Head Pose + Facial+ Vocal	36	2	41	4	0.9	0.954

(Continued)

Supplementary Table S6. *(Continued)*

Study	Sample Size (ASD/TD)	Experimental Tasks	Classification Algorithm	Modality	TP	FP	TN	FN	Sensitivity	Specificity
H. Drimalla et al., 2019	36/41	Simulated Interaction Task	Random Forest	Facial + Gaze + Vocal	24	7	34	12	0.667	0.829
			SVM		23	9	32	13	0.639	0.780
	36/40		CNN		9	14	26	27	0.25	0.650
					19	13	27	17	0.52	0.675
H. Drimalla et al., 2020	36/43	Simulated Interaction Task	Random Forest	Facial	22	12	31	14	0.611	0.721
				Gaze	19	17	26	17	0.528	0.605
				Vocal	26	10	33	10	0.722	0.767
				Facial + Gaze + Vocal	24	9	34	12	0.667	0.791
D. Alie et al., 2011	6/26	Parents and Children Interaction	VMM	Gaze	6	2	24	0	1	0.923
			HMM		2	2	24	4	0.333	0.923
N. Qiu et al., 2020	40/43	Still-Face	Naïve Bayes	Posture	30	7	36	10	0.75	0.837
					31	6	37	9	0.775	0.860
			Random Forest		31	7	36	9	0.775	0.837
					31	5	38	9	0.775	0.884
			SVM		35	10	33	5	0.875	0.767
					34	7	36	6	0.85	0.837
Z. Zhao et al., 2021	19/20	Conversation	SVM	Gaze	16	0	20	3	0.842	1
N. Kojovic et al., 2021	169/68	ADOS Assessment	CNN	Posture + Image	29	8	26	5	0.854	0.765

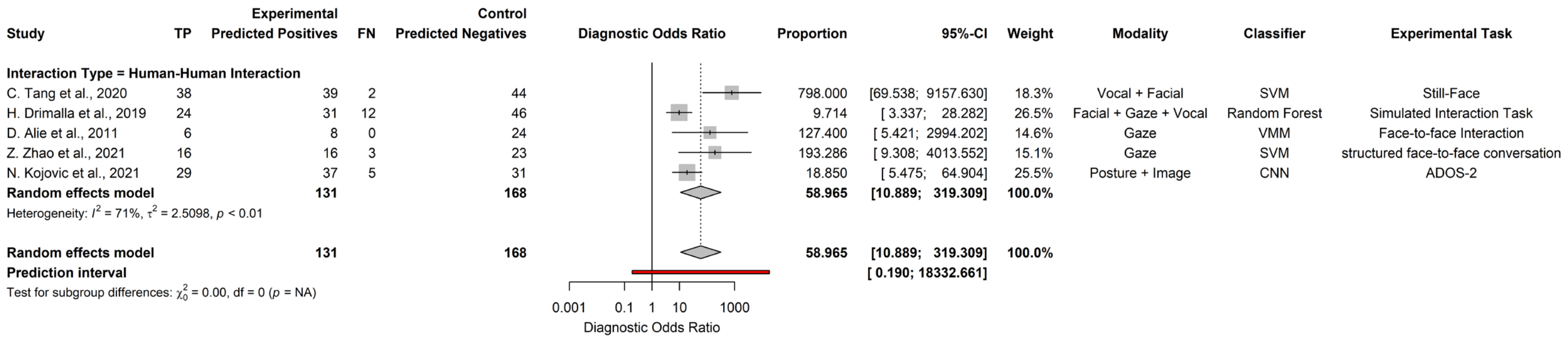

Supplementary Figure S2. Forest plot of the pooled diagnostic odds ratio of the best performing prediction outcome from each study group.

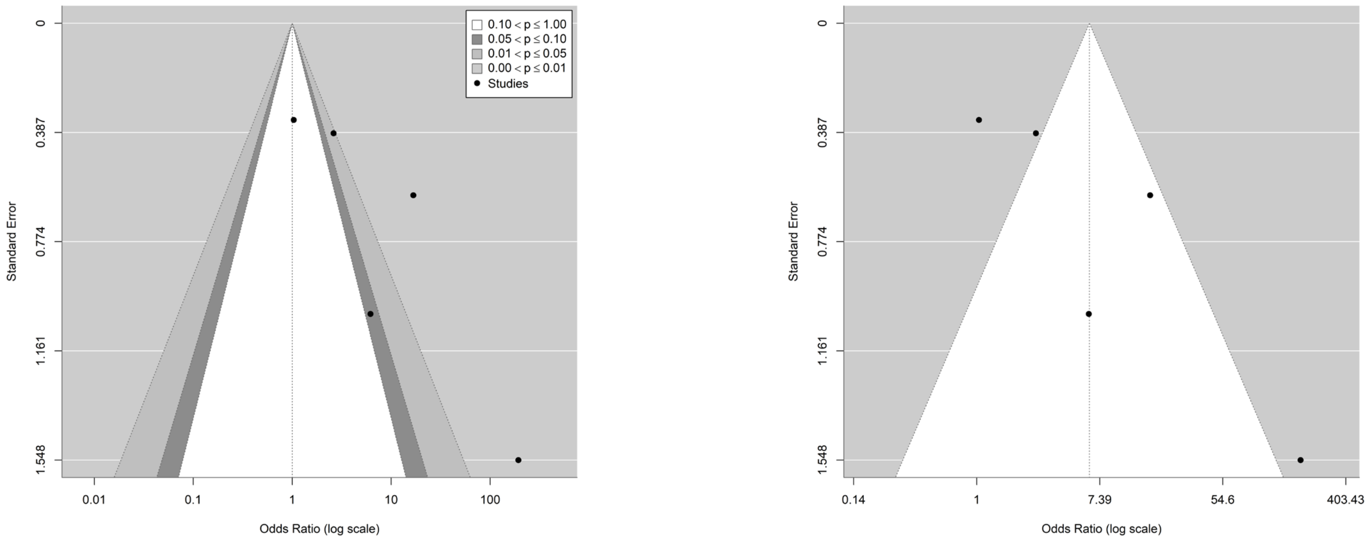

(a) (b)

Supplementary Figure S3. Funnel plots assessing publication bias: (a) before and (b) after trim-and-fill methods of aggregated outcomes from the included studies.

Supplementary Table S7. Summary of the moderator analysis on the classification algorithms reported in the diagnostic accuracy tests studies.

Moderator	Estimate	SE	z	p	95% CI
CNN	1.617	0.937	1.725	0.085	[-0.220, 3.454]
HMM	1.695	1.821	0.931	0.352	[-1.874, 5.263]
K-NN	3.197	0.893	3.580	0.0003	[1.447, 4.948]
NB	2.610	1.131	2.309	0.021	[0.394, 4.826]
RF	2.746	0.809	3.393	0.0007	[1.160, 4.332]
SVM	3.580	0.800	4.473	<0.0001	[2.011, 5.148]
VMM	4.847	2.202	2.202	0.028	[0.532, 9.162]

Supplementary Table S8. Summary of the moderator analysis on the input modalities reported in the diagnostic accuracy tests studies.

Moderator	Estimate	SE	z	p	95% CI
Facial	3.290	0.968	3.400	0.0007	[1.393, 5.187]
Facial + Gaze + Vocal	2.934	0.926	3.167	0.002	[1.118, 4.749]
Gaze	2.480	0.921	2.692	0.007	[0.674, 4.285]
Head Pose	0.425	1.054	0.403	0.687	[-1.641, 2.491]
Head Pose + Facial	3.628	1.124	3.229	0.001	[1.426, 5.830]
Head Pose + Facial + Vocal	5.080	1.185	4.288	<0.0001	[2.758, 7.402]
Head Pose + Vocal	3.468	1.090	3.181	0.002	[1.331, 5.605]
Posture	2.667	0.983	2.713	0.007	[0.741, 4.594]
Posture + Image	2.817	1.651	1.706	0.088	[-0.420, 6.053]
Vocal	3.595	0.968	3.716	0.0002	[1.699, 5.491]
Vocal + Facial	5.263	1.194	4.407	<0.0001	[2.922, 7.603]

Supplementary Table S9. Summary of the moderator analysis on the combined effect of classification algorithms and input modalities.

Moderator	Estimate	SE	z	p	95% CI
Facial	1.780	1.384	1.286	0.198	[-0.933, 4.494]
Facial + Gaze + Vocal	2.193	1.339	1.638	0.102	[-0.431, 4.816]
Gaze	0.842	1.389	0.606	0.544	[-1.880, 3.565]
Head Pose	-1.033	1.422	-0.726	0.468	[-3.821, 1.755]
Head Pose + Facial	2.149	1.473	1.459	0.145	[-0.738, 5.036]
Head Pose + Facial + Vocal	3.641	1.521	2.394	0.017	[0.660, 6.622]
Head Pose + Vocal	1.969	1.447	1.361	0.174	[-0.867, 4.805]
Posture	0.972	1.403	0.692	0.489	[-1.779, 3.721]
Posture + Image	2.817	2.117	1.331	0.183	[-1.333, 6.966]
Vocal	2.136	1.385	1.542	0.123	[-0.578, 4.851]
Vocal + Facial	3.820	1.528	2.500	0.012	[0.825, 6.815]
HMM	0.852	2.665	0.320	0.749	[-4.372, 6.076]
K-NN	1.159	0.645	1.797	0.072	[-0.105, 2.423]
NB	1.513	0.664	2.280	0.023	[0.212, 2.815]
RF	1.735	0.474	3.660	0.0003	[0.806, 2.665]
SVM	1.788	0.527	3.392	0.0007	[0.755, 2.821]
VMM	4.005	2.934	1.363	0.173	[-1.755, 9.765]

Supplementary Table S10. Summary of the moderator analysis on the combined effect of classification algorithms, input modalities and experimental tasks.

Moderator	Estimate	SE	z	p	95% CI
Facial	4.544	1.746	2.602	0.009	[1.121, 7.967]
Facial + Gaze + Vocal	4.994	1.701	2.936	0.003	[1.660, 8.328]
Gaze	3.532	1.636	2.159	0.031	[0.326, 6.738]
Head Pose	1.722	1.802	0.956	0.339	[-1.810, 5.253]
Head Pose + Facial	4.903	1.844	2.660	0.0078	[1.289, 8.517]
Head Pose + Facial + Vocal	6.396	1.881	3.400	0.0007	[2.709, 10.083]
Head Pose + Vocal	4.723	1.824	2.590	0.0096	[1.149, 8.298]
Posture	3.678	1.778	2.069	0.0385	[0.194, 7.163]
Posture + Image	2.817	0.610	4.618	<0.0001	[1.621, 4.012]
Vocal	4.898	1.751	2.797	0.005	[1.465, 8.331]
Vocal + Facial	6.575	1.887	3.484	0.0005	[2.876, 10.274]
HMM	-1.837	1.934	-0.950	0.342	[-5.628, 1.953]
K-NN	1.098	0.648	1.695	0.0902	[-0.172, 2.369]
NB	1.502	0.664	2.262	0.024	[0.200, 2.803]
RF	1.765	0.475	3.718	0.0002	[0.834, 2.695]
SVM	1.733	0.529	3.275	0.001	[0.696, 2.769]
VMM	1.316	2.296	0.573	0.567	[-3.184, 5.816]
Simulated Interaction Task	-4.773	1.667	-2.864	0.004	[-8.040, 1.507]
Still-Face	-2.387	1.695	-1.409	0.159	[-5.709, 0.934]

Study	TP	Autism Total +	TN	Control Total -	Classifier	Modality	Weight	Odds Ratio [95% CI]
**Structured Face-to-Face Conversation**								
Z. Zhao et al., 2021	16	19	20	20	SVM	Gaze	1.16%	193.29 [9.31, 4013.55]
**Still-Face Paradigm**								
N. Qiu et al., 2020	34	40	36	43	SVM	Posture	3.53%	25.83 [8.19, 81.48]
N. Qiu et al., 2020	35	40	33	43	SVM	Posture	3.55%	20.59 [6.62, 64.04]
N. Qiu et al., 2020	31	40	38	43	Random Forest	Posture	3.53%	23.21 [7.34, 73.37]
N. Qiu et al., 2020	31	40	36	43	Random Forest	Posture	3.64%	16.14 [5.54, 47.02]
N. Qiu et al., 2020	31	40	37	43	Naïve Bayes	Posture	3.59%	19.13 [6.34, 57.72]
N. Qiu et al., 2020	30	40	36	43	Naïve Bayes	Posture	3.67%	14.14 [4.93, 40.53]
C. Tang et al., 2020	36	40	41	43	KNN	Head Pose + Facial+Vocal	2.90%	134.64 [26.95, 672.75]
C. Tang et al., 2020	37	40	41	43	KNN	Vocal + Facial	2.80%	177.86 [33.08, 956.22]
C. Tang et al., 2020	26	40	41	43	KNN	Head Pose + Facial	3.14%	30.34 [7.28, 126.51]
C. Tang et al., 2020	36	40	37	43	KNN	Head Pose + Vocal	3.34%	46.79 [12.94, 169.22]
C. Tang et al., 2020	36	40	36	43	KNN	Vocal	3.38%	39.47 [11.25, 138.54]
C. Tang et al., 2020	26	40	41	43	KNN	Facial	3.14%	30.34 [7.28, 126.51]
C. Tang et al., 2020	17	40	28	43	KNN	Head Pose	3.91%	1.37 [0.57, 3.28]
C. Tang et al., 2020	38	40	42	43	SVM	Head Pose + Facial+Vocal	2.33%	436.33 [55.02, 3460.22]
C. Tang et al., 2020	38	40	42	43	SVM	Vocal + Facial	2.33%	436.33 [55.02, 3460.22]
C. Tang et al., 2020	34	40	41	43	SVM	Head Pose + Facial	3.01%	88.11 [19.14, 405.65]
C. Tang et al., 2020	36	40	36	43	SVM	Head Pose + Vocal	3.38%	39.47 [11.25, 138.54]
C. Tang et al., 2020	36	40	37	43	SVM	Vocal	3.34%	46.79 [12.94, 169.22]
C. Tang et al., 2020	34	40	40	43	SVM	Facial	3.21%	61.42 [15.51, 243.28]
C. Tang et al., 2020	36	40	13	43	SVM	Head Pose	3.50%	3.59 [1.11, 11.57]
**Simulated Interaction Task**								
H. Drimalla et al., 2020	24	36	34	43	Random Forest	Facial + Gaze + Vocal	3.54%	7.12 [2.65, 19.15]
H. Drimalla et al., 2020	26	36	33	43	Random Forest	Vocal	3.53%	8.05 [2.98, 21.79]
H. Drimalla et al., 2020	19	36	26	43	Random Forest	Gaze	3.67%	1.69 [0.70, 4.08]
H. Drimalla et al., 2020	22	36	31	43	Random Forest	Facial	3.61%	3.91 [1.54, 9.91]
H. Drimalla et al., 2019	19	36	27	40	CNN	Facial + Gaze + Vocal	3.63%	2.27 [0.91, 5.68]
H. Drimalla et al., 2019	9	36	26	40	CNN	Facial + Gaze + Vocal	3.56%	0.63 [0.24, 1.68]
H. Drimalla et al., 2019	23	36	32	41	SVM	Facial + Gaze + Vocal	3.55%	5.96 [2.22, 15.95]
H. Drimalla et al., 2019	24	36	34	41	Random Forest	Facial + Gaze + Vocal	3.48%	9.02 [3.18, 25.58]
**Parents and Child Interaction**								
D. Alie et al., 2011	2	6	24	26	HMM	Gaze	1.77%	5.44 [0.72, 41.07]
D. Alie et al., 2011	6	6	24	26	VMM	Gaze	1.20%	127.40 [5.42, 2994.20]
**ADOS-2 Assessment**								
N. Kojovic et al., 2021	29	34	26	34	CNN	Posture + Image	2.07%	16.72 [5.07, 55.18]
RE Model (Q = 186.42, df = 31, p <0.0001)							100%	15.92 [4.78, 53.06]

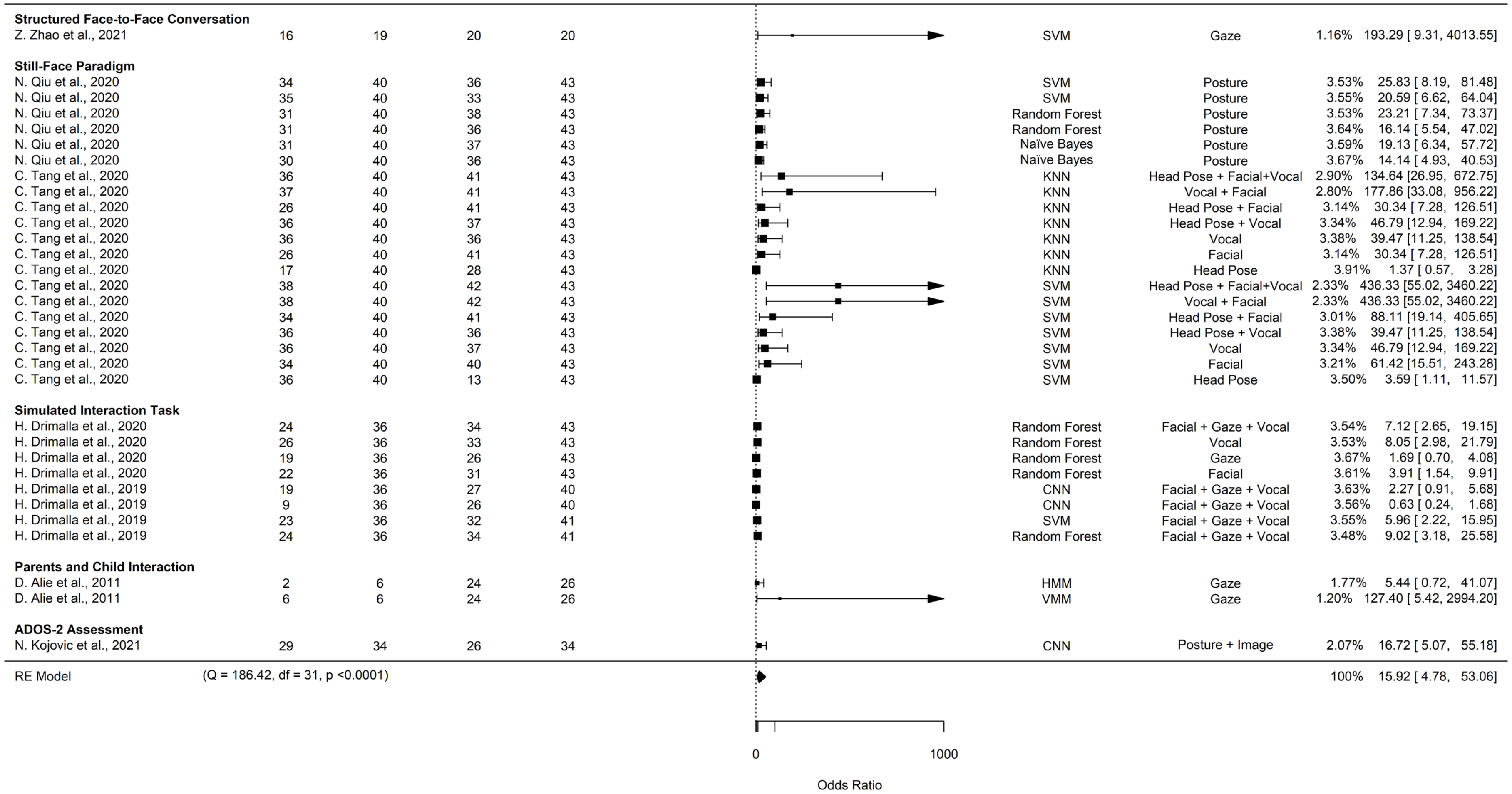

Supplementary Figure S4. Forest plot of three-level meta-regression grouped by study groups.

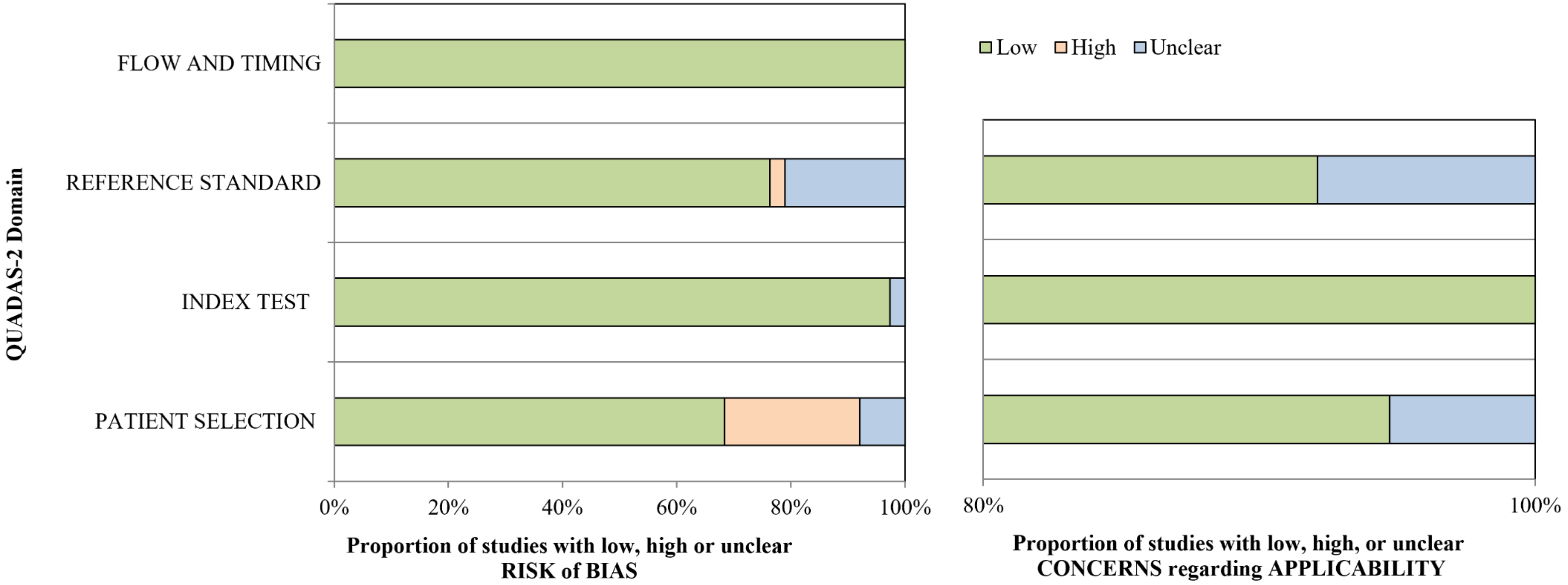

Supplementary Figure S5. Quality assessment of the eligible studies by the QUADAS-2 checklist.

Supplementary Table S11. Quality assessment details of the eligible studies.

Study	Patient Selection						Index Test				Reference Standard				Flow and Timing				
	PS-Q1	PS-Q2	PS-Q3	ROB	PS-Q4	AC	IT-Q1	IT-Q2	ROB	AC	RS-Q1	RS-Q2	ROB	AC	FAT-Q1	FAT-Q2	FAT-Q3	FAT-Q4	ROB
A. Di Nuovo et al., 2018	Y	Y	Y	L	Y	L	N	N	L	L	Y	Y	L	L	Y	Y	Y	Y	L
C. Eunji et al., 2017	Y	N	Y	L	Y	L	N	Y	L	L	Y	Y	L	L	Y	Y	Y	Y	L
C. Tang et al., 2020[c]	Y	N	Y	L	Y	L	N	Y	L	L	Y	Y	L	L	Y	Y	Y	Y	L
E. Chong et al., 2017	Y	N	Y	L	Y	L	N	N	L	L	Y	Y	L	L	Y	NA	NA	Y	L
G. Nie et al., 2018	Y	N	Y	NA	Y	L	N	Y	L	L	Y	Y	L	L	Y	NA	NA	NA	L
H. Drimalla et al., 2019[c]	Y	N	Y	L	Y	L	N	Y	L	L	Y	Y	L	L	Y	Y	Y	Y	L
H. Drimalla et al., 2020[c]	Y	N	Y	L	Y	L	N	Y	L	L	Y	Y	L	L	Y	Y	Y	Y	L
H. Javed et al., 2020	Y	N	NA	H	Y	L	N	N	L	L	NA	Y	NA	NA	NA	NA	NA	Y	L
K. S. Lohan et al., 2018	Y	N	Y	L	Y	L	N	N	L	L	Y	Y	L	L	Y	Y	Y	N	L
E. Chong et al., 2020	Y	N	Y	L	Y	L	N	Y	L	L	Y	Y	L	L	Y	Y	Y	N	L
D. Alie et al., 2011[c]	Y	N	Y	H	Y	L	N	N	L	L	Y	Y	L	L	Y	Y	Y	Y	L
N. Zhang et al., 2022	Y	Y	Y	L	Y	L	N	Y	L	L	Y	Y	L	L	Y	Y	Y	Y	L
N. Qiu et al., 2020[c]	Y	N	Y	L	Y	L	N	N	L	L	Y	Y	L	L	Y	Y	Y	Y	L
W. B. Liu et al., 2017	Y	N	NA	L	Y	L	N	N	L	L	Y	Y	L	L	Y	Y	Y	Y	L

(Continued)

Supplementary Table S11. *(Continued)*

Study	Patient Selection						Index Test				Reference Standard				Flow and Timing				
	PS-Q1	PS-Q2	PS-Q3	ROB	PS-Q4	AC	IT-Q1	IT-Q2	ROB	AC	RS-Q1	RS-Q2	ROB	AC	FAT-Q1	FAT-Q2	FAT-Q3	FAT-Q4	ROB
M. Varma et al., 2022	Y	N	Y	H	Y	L	N	Y	L	L	NA	Y	NA	L	Y	NA	NA	Y	L
J. Zhang et al., 2022	Y	N	Y	L	Y	L	N	Y	L	L	Y	Y	L	L	Y	Y	Y	Y	L
J. T. Megerian et al., 2022	Y	N	Y	H	Y	L	N	Y	L	L	Y	Y	NA	L	NA	N	NA	N	L
M. Alcaniz et al., 2022	Y	Y	Y	L	Y	L	N	N	L	L	Y	Y	L	L	Y	Y	Y	Y	L
I. Akin-Bulbul et al., 2022	Y	N	Y	L	Y	L	N	N	L	L	Y	Y	L	L	Y	Y	Y	Y	L
A. Adham et al., 2022	NA	N	NA	H	Y	L	N	Y	L	L	NA	Y	NA	NA	NA	NA	NA	N	L
Z. Zhao et al., 2021[c]	Y	N	Y	L	Y	L	N	Y	L	L	Y	Y	L	L	Y	Y	Y	Y	L
G. Zhang et al., 2021	Y	Y	Y	L	Y	L	N	Y	L	L	Y	Y	L	L	Y	Y	Y	Y	L
N. Kojovic et al., 2021[c]	Y	N	Y	L	Y	L	N	N	L	L	Y	Y	L	L	Y	Y	Y	Y	L
C. Wu et al., 2021	Y	Y	Y	H	Y	NA	N	N	L	L	Y	Y	NA	L	NA	NA	NA	Y	L
D. Anagnostopoulou et al., 2021	Y	N	NA	H	Y	L	N	N	L	L	Y	Y	L	L	Y	Y	Y	Y	L
J. Li et al., 2021	Y	Y	Y	L	Y	L	N	Y	L	L	Y	Y	L	L	Y	Y	Y	N	L
P. R. Krishnappababu, et al., 2021	Y	N	Y	H	Y	L	N	N	L	L	Y	Y	L	L	Y	Y	Y	Y	L

(Continued)

Supplementary Table S11. *(Continued)*

Study	Patient Selection						Index Test				Reference Standard				Flow and Timing				
	PS-Q1	PS-Q2	PS-Q3	ROB	PS-Q4	AC	IT-Q1	IT-Q2	ROB	AC	RS-Q1	RS-Q2	ROB	AC	FAT-Q1	FAT-Q2	FAT-Q3	FAT-Q4	ROB
C. Ko et al., 2023	Y	N	Y	L	Y	L	N	Y	L	L	Y	Y	L	L	Y	Y	Y	N	L
V. G. Prakash et al., 2023	Y	Y	NA	NA	Y	NA	NA	Y	NA	L	NA	NA	NA	L	NA	NA	NA	NA	L
Krishnappababu et al., 2023a	*Y*	*N*	*Y*	*L*	*Y*	*L*	*N*	*Y*	*L*	*L*	*Y*	*Y*	*L*	*L*	*Y*	*Y*	*Y*	*N*	*L*
Krishnappababu et al., 2023b	Y	N	Y	L	Y	L	N	N	L	L	Y	Y	L	L	Y	Y	Y	Y	L
J. Liu et al., 2023	Y	Y	NA	L	Y	L	N	N	L	L	Y	Y	NA	L	Y	Y	Y	Y	L
Minissi, M. E. et al., 2024	Y	N	Y	L	Y	L	N	Y	L	L	Y	Y	L	L	Y	Y	Y	Y	L
W. Saakyan et al., 2023	Y	N	Y	Y	Y	L	N	N	L	L	Y	Y	L	L	Y	Y	Y	Y	L
D. Q. McDonald et al.,2023	Y	N	Y	L	Y	L	N	N	L	L	Y	Y	L	L	Y	Y	Y	Y	L
Z. Zhao et al., 2024	NA	Y	NA	NA	Y	L	N	N	L	L	NA	Y	NA	NA	NA	NA	NA	Y	L
X. Wang et al., 2024	Y	N	Y	L	Y	L	N	N	L	L	Y	Y	L	L	Y	Y	Y	Y	L
L. L. Liu et al.,2024	Y	N	Y	H	Y	L	N	N	L	L	Y	Y	H	L	Y	Y	Y	Y	L

Patient Selection, PS; Index Test, IT; Reference Standards: RS; Flow and Timing: FAT; Risk of Bias, ROB; Applicability Concerns, AC; YES, Y; NO, N; LOW, L; HIGH, H; UNCLEAR, NA; [c], studies included in the meta-analysis.
